# Lightweight Design of Variable-Stiffness Cylinders with Reduced Imperfection Sensitivity Enabled by Continuous Tow Shearing and Machine Learning

**DOI:** 10.3390/ma15124117

**Published:** 2022-06-09

**Authors:** Rogério R. dos Santos, Saullo G. P. Castro

**Affiliations:** 1 Division of Mechanical Engineering, Aeronautics Institute of Technology, São José dos Campos 12228-900, Brazil; rsantos9@gmail.com; 2 Faculty of Aerospace Engineering, Delft University of Technology, 2629 HS Delft, The Netherlands

**Keywords:** buckling, post-buckling, imperfection, imperfection sensitivity, filament winding, support vector machine, Kriging, random forest, differential evolution

## Abstract

The present study investigates how to apply continuous tow shearing (CTS) in a manufacturable design parameterization to obtain reduced imperfection sensitivity in lightweight, cylindrical shell designs. The asymptotic nonlinear method developed by Koiter is applied to predict the post-buckled stiffness, whose index is constrained to be positive in the optimal design, together with a minimum design load. The performance of three machine learning methods, namely, Support Vector Machine, Kriging, and Random Forest, are compared as drivers to the optimization towards lightweight designs. The new methodology consists of contributions in the areas of problem modeling, the selection of machine learning strategies, and an optimization formulation that results in optimal designs around the compromise frontier between mass and stiffness. The proposed ML-based framework proved to be able to solve the inverse problem for which a target design load is given as input, returning as output lightweight designs with reduced imperfection sensitivity. The results obtained are compatible with the existing literature where hoop-oriented reinforcements were added to obtain reduced imperfection sensitivity in composite cylinders.

## 1. Introduction

The buckling performance of cylindrical shells is recognized to be highly sensitive to geometric, load, and material imperfections, a behavior that was identified already since the first attempts to correlate experimental with theoretical predictions. The use of straight-fiber laminated composite materials has enabled the maximization of the mass-normalized load-carrying capacity of these shells, while customarily applying conservative knock-down factors to reduce the theoretical load-carrying capacity. Recent studies present measured imperfections for filament-wound composite cylinders [1], showing a thickness pattern that highly depends on the design and that significantly affects the buckling strength. Furthermore, other defects, such as those created during service due to low-velocity impacts [2,3,4], can also lead to a significant knock-down on the theoretical performance of cylindrical shells.

The application of novel composite manufacturing technologies such as continuous tow shearing (CTS) can enable a larger design space by means of fiber steering and thickness variation. This larger design space encompasses composite cylindrical shell designs that showed an imperfection-insensitive behavior and which have the potential to undergo buckling without collapse.

Imperfection-sensitive structures such as cylindrical shells are known for their large number of nonlinear paths and nearby bifurcation points. During the experimental evaluation of such structures, only one nonlinear path is preferably chosen according to the localization event produced by geometric, load, and material imperfections. The high sensitivity of the chosen nonlinear path with respect to this localization event was called, by Thomson and Virgin, “spatial chaos” [5], justifying the large scatter of experimental data that is usually obtained from the experimental assessment of cylindrical shells, and other imperfection-sensitive shells.

Recently, Groh et al. [6] proposed a representative and simple model based on rigid links supported by transverse springs to numerically demonstrate the concept of spatial chaos, where small changes in the geometric imperfections result in a large scatter of the maximum load-carrying capacity. The NASA-8007 guideline [7], which is derived from the collection of experimental results from Seide, Weingarten, and Morgan [8,9], reports a large number of experimental results for cylindrical shells having different radius-per-thickness ratios, where the aforementioned scatter can be clearly observed. The guideline defines a lower-bound curve with respect to the scattered data that is frequently used to calculate knock-down factors (KDF), and these KDF can be directly used in combination with the theoretical linear buckling load of cylindrical shells, as discussed by Hilburger [10]. Such a resource is useful while designing shells for a given required compression load and while taking the imperfection sensitivity into account.

The determination of the post-buckling behavior of shells requires nonlinear analysis schemes that usually fall into one of three categories: analytical, axisymemtric, or full 3D models. The asymptotic theory originally proposed by Koiter [11] allows for a rapid evaluation of the initial post-buckling behavior of structures, and the method has been used within semi-analytical contexts [12,13,14], and mostly within finite element schemes [15,16,17,18,19,20,21,22,23,24,25]. In recent years, the method has been applied in the context of the variable stiffness of panel-type structures [21,23,26,27,28,29], and in the analysis of imperfection-sensitive shells [25,30,31,32,33].

White et al. [34] proposed the use of Koiter’s asymptotic method to achieve reduced imperfection sensitivity in variable-stiffness cylindrical panels, solving the numerical problem by means of generalized differential and integral quadrature. A genetic algorithm optimizer was used to find optimal straight- and curved-fiber composite layouts for the curved panels under investigation. The best post-buckling performance achieved by the curved-fiber designs showed virtually no drop in axial stiffness while entering the post-buckling regime. The work of White et al. [34] inspired the present study on the lightweight design of cylindrical shells, aiming at reduced imperfection sensitivity.

The main scientific contributions of the present study are:

- a new parameterization scheme for variable-stiffness cylindrical shells manufactured using continuous tow shearing (CTS), which result in a vast design space that is explored in order to attain designs with reduced imperfection sensitivity;
- the comparison of the performance of three classes of machine learning strategies with multiple kernel models, in a total of eighteen distinct instances;
- an algorithm to calculate the optimal design that meets the target levels of mass, buckling load, and post-buckling stiffness. The inverse problem is formulated within the scope of global optimization for mixed-integer variables.

The study is concluded by showing the potential of the algorithm to solve the inverse problem related to the proposed design scheme, where for each design load, the optimal design that is lightweight and has a reduced imperfection sensitivity can be obtained efficiently.

The paper is organized as follows. Section 2 presents the characteristics of the computational model of a cylinder and explains the key features considered in the analysis. The linear buckling is discussed in Section 3, and the asymptotic expansion is discussed in Section 4. Section 5 outlines the characteristics of the Support Vector Machine, Kriging, and Random Forest algorithms, and presents the results obtained from the adjustment of the kernel parameters. Section 6 details the proposed algorithm that computes the inverse problem and discusses the results obtained from numerical experiments. The conclusions are summarized in Section 7.

## 2. Design Parameterization with Circumferentially Oriented Thickness Patterns

The proposed parameterization consists of circumferentially oriented thickness patterns created from the nonlinear steer–thickness coupling that is enabled by the continuous tow shearing (CTS) manufacturing process.

**Figure 1:**
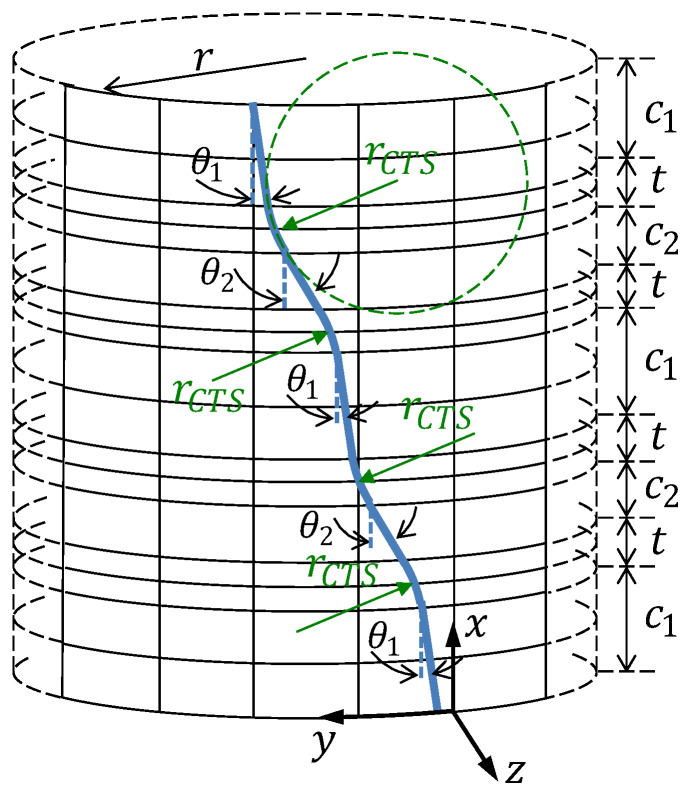
Design parameterization based on circumferentially oriented thickness patterns.

Figure 1 schematically shows the design parameterization in an example with two regions $c_{2}$ (n=2) and three regions $c_{1}$. Between the regions $c_{1}$ and $c_{2}$, there is a transition region *t*. The tow angle θ(x) at the regions $c_{1}$ and $c_{2}$ is, respectively, $\theta_{1}$ and $\theta_{2}$. In the continuous tow shearing (CTS) process, the robot head moves in such a way that the deposited tow width remains constant, such that any tow angle different than zero will generate a tow of higher thickness, where the local thickness can be calculated with

(1) $$h\left(x\right)=h_{tow}/\cos\theta (x)$$

as shown in [35,36,37,38]. This is true if we assume that the shift direction of the deposition head, where it moves to start the next tow, is the circumferential direction of the cylinder. The transition regions *t* that are located between the two regions $c_{1}$ and $c_{2}$ are made by steering the fibers with a defined steering radius $r_{CTS}\geq {r_{CTS}}_{min}$, being ${r_{CTS}}_{min}=50$ mm [35], as inspired by the work of Ummels and Castro [39] on overlap-stiffened structures suggesting pre-determined steering angle values. In the present study, it is assumed that the fiber angles in the transition region change linearly with *x*. Hence, the length *t* can be calculated as

(2) $$t=r_{CTS}\sin\left(\left|\theta_{c_{2}}-\theta_{c_{1}}\right|\right).$$

If *n* regions with length $c_{2}$ are used between n+1 regions with length $c_{1}$, the following simple equation for the total cylinder length *L* can be derived:

(3) $$L=\left(2t+c_{2}\right)n+c_{1}\left(n+1\right).$$

A nondimensional quantity ${c_{2}}_{ratio}$ can be conveniently used to express the ratio between regions $c_{1}$ and $c_{2}$:

(4) $${c_{2}}_{ratio}=\frac{c_{2}}{{c_{2}}_{max}}$$

such that $0\leq {c_{2}}_{ratio}\leq 1$. The value ${c_{2}}_{max}$ is the maximum length of region $c_{2}$, and is determined assuming $c_{1}=0$ in Equation (3), leading to

(5) $${c_{2}}_{max}=\frac{L-2tn}{n}.$$

Assuming that the whole cylinder length is covered with transition regions *t*, implying $c_{1}=c_{2}=0$, the maximum number of $c_{2}$ regions, or the maximum value for *n*, can be calculated for the proposed design parameterization as

(6) $$n_{max}=\frac{L}{2t}.$$

The special case ${c_{2}}_{ratio}=0$ and n>0 leads to a feasible design consisting of a combination of regions $c_{1}$ and transition regions *t*. The case with ${c_{2}}_{ratio}=1$ and n>0 also leads to a feasible design combining transition regions *t* with regions $c_{2}$. When ${c_{2}}_{ratio}=0$ and n=0, a constant-stiffness cylinder with stacking sequence $\pm \theta_{1}$ is generated, whereas ${c_{2}}_{ratio}=1$ and n=0 leads to a constant-stiffness cylinder with stacking sequence $\pm \theta_{2}$.

In summary, the proposed parameterization takes, as input:

- *n*: the number of regions $c_{2}$;
- $r_{CTS}$: the CTS steering radius in the transition zone;
- ${c_{2}}_{ratio}$: the ratio between the design length $c_{2}$ with respect to ${c_{2}}_{max}$, which is calculated as per Equation (5);
- $\theta_{1}$ and $\theta_{2}$: the respective angles at regions $c_{1}$ and $c_{2}$.

With these inputs, the following design parameters are calculated:

- The length of the transition regions *t*, according to Equation (2);
- The length $c_{2}$, based on ${c_{2}}_{ratio}$ and ${c_{2}}_{max}$;
- The length $c_{1}$, according to Equation (3).

Furthermore, the adopted strategy to control the finite element mesh size takes, as input:

- ${n_{x}}_{t}$: the number of nodes along *x*, along the length of each transition zone, given by *t*;
- $n_{y}$: the number of nodes along the circumferential direction, which is constant along the length. If $n_{y}$ is not given, then it is calculated assuming a desired aspect ratio of $\frac{n_{y}}{n_{x}}=1$;
- the maximum aspect ratio of the elements in the circumferential direction, with respect to the axial direction:
  (7) $${\left(\frac{n_{y}}{n_{x}}\right)}_{max}.$$
  If this value is exceeded, more nodes in the *x* direction are added to the regions $c_{1}$ and $c_{2}$ to keep
  (8) $$\frac{n_{y}}{n_{x}}\leq {\left(\frac{n_{y}}{n_{x}}\right)}_{max}.$$
  Note that Equation (7) does not affect the transition regions with length *t*, which have their number of nodes along the length determined by ${n_{x}}_{t}$.

With these mesh inputs, the number of nodes along the lengths $c_{1}$ and $c_{2}$ are then calculated. The workflow is shown in Algorithm 1.

The proposed design parameterization creates a tailored circumferentially oriented thickness pattern, and enables the proposition of an optimization formulation. Setting the main objective as a lightweight design aiming at minimum mass *m*, with a given design load $P_{design}$, a positive post-buckling stiffness $b_{I}>0$, and the linear buckling load of the cylinder $P_{critical}$, the resulting optimization problem can be formally stated as

(9) $$\min_{\left(r_{CTS},n,{c_{2}}_{ratio},\theta_{1},\theta_{2}\right)}m$$

subject to

(10) $$\begin{array}{c} P_{critical}\geq P_{design}\\ b_{I}>0\\ r_{CTS}\geq {r_{CTS}}_{min}\\ 1\leq n\leq n_{max}\\ 0\leq {c_{2}}_{ratio}\leq 1\\ 0\leq \theta_{1}\leq 75\\ 0\leq \theta_{2}\leq 75 \end{array}$$

**Algorithm 1:** The modeling phase /* Initial setup*/ n← number of regions $c_{2}$; $r_{CTS}\leftarrow$ steering radius; ${c_{2}}_{ratio}\leftarrow$ ratio in agreement with Equation (5);
 $\theta_{1}\leftarrow$ tow angle at $c_{1}$;
 $\theta_{2}\leftarrow$ tow angle at $c_{2}$;
 /* Intermediary computation */ t← length of the transition regions (Equation (2));
 $c_{2}\leftarrow$ length (Equations (4) and (5));
 $c_{1}\leftarrow$ length (Equation (3)); Ensure $\frac{n_{y}}{n_{x}}\leq {\left(\frac{n_{y}}{n_{x}}\right)}_{max}$;

 Proceed with model evaluation;
 /* Output*/ Return the number of nodes along the lengths $c_{1}$ and $c_{2}$.


Although the formulation expressed by Equations (9) and (10) may lead to optimal designs, there is no guarantee that a feasible design exists for an arbitrary $P_{design}$ and $b_{I}>0$. Therefore, a new multi-objective formulation is proposed:

(11) $$\min_{\left(r_{CTS},n,{c_{2}}_{ratio},\theta_{1},\theta_{2}\right)}\left\{\alpha_{1}{\left(m-m_{ref}\right)}^{2}+\alpha_{2}{\left(P_{critical}-P_{ref}\right)}^{2}+\alpha_{3}{\left(b_{I}-b_{ref}\right)}^{2}\right\}$$

subject to

(12) $$\begin{array}{c} 0.05\leq r_{CTS}\leq 0.20\\ 1\leq n\leq n_{max}\\ 0\leq {c_{2}}_{ratio}\leq 1\\ 0\leq \theta_{1}\leq 75\\ 0\leq \theta_{2}\leq 75 \end{array}$$

where $m_{ref},P_{ref}$, and $b_{ref}$ are reference values for $m,P_{critical}$, and $b_{I}$, respectively. The weighting values $\alpha_{1},\alpha_{2}$, and $\alpha_{3}$ represent the preference in optimizing each criteria.

An advantage of this formulation (Equations (11) and (12)) is the ability to contemplate some constraints as objectives to be minimized. It avoids the issue that the set of inequalities (Equation (10)) may, in some circumstances, lead to an empty set of solutions. Furthermore, the optimization process will provide feasible designs even when an infeasible set of reference values $m_{ref},P_{ref}$ and $b_{ref}$ is given.

Although finding suitable reference values for the new objective function (Equation (11)) is not trivial, the current proposition consists in the usage of machine learning strategies as a heuristic to provide useful estimates. Those parameters will then be used as part of a global optimization procedure, in the domain of mixed-integer variables, which will refine the search of the global optimum.

Further details on this topic can be found in Section 6.

## 3. Linear Buckling Constraint

The linear buckling load $P_{critical}$ is compared to the design load $P_{design}$ in the optimization scheme described by Equation (10). In order to calculate the linear buckling load of the proposed variable-stiffness design, the single-curvature Bogner–Fox–Schmit–Castro (SC-BFSC) finite element is selected. First presented by Wang et al. [40], the SC-BFSC element is based on the plate version presented by Castro and Jansen [24], which is a C1-contiguous and confirming element obtained by taking tensor products of cubic Hermite splines [41]. With 4 nodes per element and 10 degrees of freedom per node, the BFSC approximates the in-plane and out-of-plane displacements using third-order polynomials, leading to a fast convergence for cases with variable stiffness.

**Figure 2:**
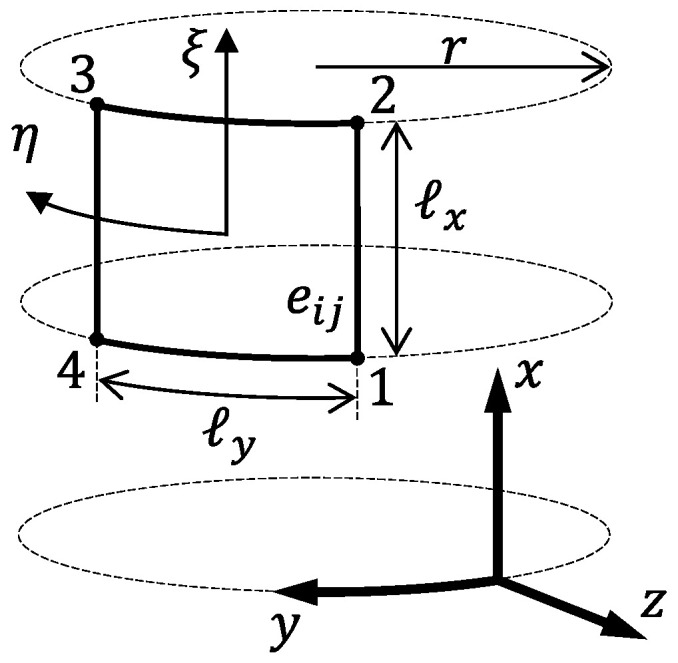
Single-curvature BFSC element and the global coordinate system xyz.

Figure 2 illustrates a SC-BFSC element and the global coordinate system xyz used, where coordinate *y* is curvilinear following the circumferential perimeter, such that at y=2πr, the path closes on itself. The displacements along x,y,z are, respectively, u,v,w, and they are approximated within each element as:

(13) $$\begin{array}{c} u_{e},v_{e},w_{e}=\sum_{i=1}^{4}S_{i}^{u,v,w}{u_{e}}_{i} \end{array}$$

where $S_{i}^{u,v,w}$ and ${u_{e}}_{i}$ are the shape functions and the 10 degrees of freedom of the *i*th node of the SC-BFSC element, being in the following order: *u*, ∂u/∂x, ∂u/∂y, *v*, ∂v/∂x, ∂v/∂y, *w*, ∂w/∂x, ∂w/∂y, $\partial^{2}w/\partial x\partial y$. For the SC-BFSC element, the same shape functions of the plate BFSC element [24] can be used:

(14) $$\begin{array}{c} S_{i}^{u}=\left[\begin{array}{cccccccccc} H_{i} & H_{i}^{x} & H_{i}^{y} & 0 & 0 & 0 & 0 & 0 & 0 & 0 \end{array}\right]\\ S_{i}^{v}=\left[\begin{array}{cccccccccc} 0 & 0 & 0 & H_{i} & H_{i}^{x} & H_{i}^{y} & 0 & 0 & 0 & 0 \end{array}\right]\\ S_{i}^{w}=\left[\begin{array}{cccccccccc} 0 & 0 & 0 & 0 & 0 & 0 & H_{i} & H_{i}^{x} & H_{i}^{y} & H_{i}^{xy} \end{array}\right] \end{array}$$

with the cubic Hermite functions $H_{i}$, $H_{i}^{x}$, $H_{i}^{y}$, $H_{i}^{xy}$ calculated using natural coordinates [42,43,44]:

(15) $$\begin{array}{c} H_{i}=\frac{1}{16}{\left(\xi +\xi_{i}\right)}^{2}\left(\xi \xi_{i}-2\right){\left(\eta +\eta_{i}\right)}^{2}\left(\eta \eta_{i}-2\right)\\ H_{i}^{x}=-\frac{\ell_{x}}{32}\xi_{i}{\left(\xi +\xi_{i}\right)}^{2}\left(\xi \xi_{i}-1\right){\left(\eta +\eta_{i}\right)}^{2}\left(\eta \eta_{i}-2\right)\\ H_{i}^{y}=-\frac{\ell_{y}}{32}{\left(\xi +\xi_{i}\right)}^{2}\left(\xi \xi_{i}-2\right)\eta_{i}{\left(\eta +\eta_{i}\right)}^{2}\left(\eta \eta_{i}-1\right)\\ H_{i}^{xy}=\frac{\ell_{x}\ell_{y}}{64}\xi_{i}{\left(\xi +\xi_{i}\right)}^{2}\left(\xi \xi_{i}-1\right)\eta_{i}{\left(\eta +\eta_{i}\right)}^{2}\left(\eta \eta_{i}-1\right) \end{array}$$

where $\ell_{x},\ell_{y}$ are, respectively, the finite element dimensions along x,y, as represented in Figure 2. Using the proposed nodal connectivity for the SC-BFSC element, the nodal degrees of freedom ${u_{e}}_{i}$ and the respective shape functions $S_{i}^{u,v,w}$ are concatenated as:

(16) $$\begin{array}{c} u_{e}={\left\{\begin{array}{cccc} {u_{e}}_{1} & {u_{e}}_{2} & {u_{e}}_{3} & {u_{e}}_{4} \end{array}\right\}}^{⊤}\\ S^{u}=\left[\begin{array}{cccc} S_{1}^{u} & S_{2}^{u} & S_{3}^{u} & S_{4}^{u} \end{array}\right]\\ S^{v}=\left[\begin{array}{cccc} S_{1}^{v} & S_{2}^{v} & S_{3}^{v} & S_{4}^{v} \end{array}\right]\\ S^{w}=\left[\begin{array}{cccc} S_{1}^{w} & S_{2}^{w} & S_{3}^{w} & S_{4}^{w} \end{array}\right] \end{array}$$

with $S^{u}$, $S^{v}$, and $S^{w}$ being matrices of shape 1×40.

The total potential energy of the cylindrical shell under axial compression can be represented by Equation (17), where Ω represents the shell domain and δΩ the shell boundaries, i.e., the loaded edges. The first term represents the strain energy based on equivalent single-layer theories [45,46], while the second term represents the work conducted by external forces at the boundaries $\hat{N}$, multiplied by a scalar λ, with $\hat{N}$ expressed in force per length units.

(17) $$\phi =\frac{1}{2}\int_{\Omega }\left(N\varepsilon +M\kappa \right)d\Omega -\int_{\delta \Omega }\lambda {\hat{N}}^{⊺}ud\left(\delta \Omega \right)$$

The strain energy functional of the entire shell continuum is built from the individual contributions of all finite elements $\phi_{e}$:

(18) $$\phi_{e}=\frac{1}{2}\int_{y=y_{1}}^{y_{4}}\int_{x=x_{1}}^{x_{2}}\left(N\varepsilon +M\kappa \right)dxdy$$

where the membrane forces are $N={\left\{N_{xx},N_{yy},N_{xy}\right\}}^{⊤}$ and the distributed moments are $M={\left\{M_{xx},M_{yy},M_{xy}\right\}}^{⊤}$. The integration limits $x_{1}\leq x\leq x_{2}$ and $y_{1}\leq y\leq y_{4}$ define the domain of one finite element $\Omega_{e}$. The membrane ε and rotational κ strains are assumed to follow Sanders-type kinematics [47], also discussed in references [48,49] for cylindrical shells, and in references [50,51,52,53] for conical shells:

(19) $$\begin{array}{c} \varepsilon =\left\{\begin{array}{c} \varepsilon_{xx}\\ \varepsilon_{yy}\\ \gamma_{xy} \end{array}\right\}=\left\{\begin{array}{c} {u,}_{x}+\frac{1}{2}{w,}_{x}^{2}\\ {v,}_{y}+\frac{1}{r}w+\frac{1}{2}{w,}_{y}^{2}+\frac{1}{2}\frac{1}{r^{2}}v^{2}-\frac{1}{r}v{w,}_{y}\\ {u,}_{y}+{v,}_{x}+{w,}_{x}{w,}_{y}-\frac{1}{r}v{w,}_{x} \end{array}\right\}\\ \kappa =\left\{\begin{array}{c} \kappa_{xx}\\ \kappa_{yy}\\ \kappa_{xy} \end{array}\right\}=\left\{\begin{array}{c} -{w,}_{xx}\\ -{w,}_{yy}+\frac{1}{r}{v,}_{y}\\ -2{w,}_{xy}+\frac{1}{r}{v,}_{x} \end{array}\right\} \end{array}$$

with ${(\cdot ),}_{x}=\partial \left(\cdot \right)/\partial x$ used as a compact notation for partial derivatives.

At the bifurcation point, the following state of equilibrium exists, considering all $n_{e}$ elements:

(20) $$\begin{array}{c} \delta \phi =\sum_{e=1}^{n_{e}}\delta \phi_{e}-\int_{\delta \Omega }\lambda {\hat{N}}^{⊺}\delta ud\left(\delta \Omega \right)=\\ \left[\sum_{e=1}^{n_{e}}\int_{\Omega_{e}}\left(N^{⊤}\delta \varepsilon +M^{⊤}\delta \kappa \right)d\Omega_{e}\right]\\ -\int_{\delta \Omega }\lambda {\hat{N}}^{⊺}\delta ud\left(\delta \Omega \right)=0 \end{array}$$

Expressing the displacements within one element in terms of nodal coordinates $u_{e}$, as in Equation (13), δε and δκ can be calculated from Equation (19) as:

(21) $$\begin{array}{c} \delta \varepsilon =B_{m}\delta u_{e}\\ \delta \kappa =B_{b}\delta u_{e} \end{array}$$

where $B_{m}$ and $B_{b}$ are defined as:

$$B_{m}=\left[\begin{array}{c} S_{,x}^{u}+{w,}_{x}S_{,x}^{w}\\ S_{,y}^{v}+\frac{1}{r}S^{w}+{w,}_{y}S_{,y}^{w}+\frac{1}{r^{2}}vS^{v}-\frac{1}{r}vS_{,y}^{w}-\frac{1}{r}{w,}_{y}S^{v}\\ S_{,y}^{u}+S_{,x}^{v}+{w,}_{x}S_{,y}^{w}+{w,}_{y}S_{,x}^{w}-\frac{1}{r}vS_{,x}^{w}-\frac{1}{r}{w,}_{x}S^{v} \end{array}\right]$$

$$B_{b}=\left[\begin{array}{c} -S_{,xx}^{w}\\ -S_{,yy}^{w}+\frac{1}{r}S_{,y}^{v}\\ -2S_{,xy}^{w}+\frac{1}{r}S_{,x}^{v} \end{array}\right]$$

noting that the partial derivatives of $S^{u,v,w}$ are directly calculated from the shape functions of Equation (14) in terms of the natural coordinates ξ,η, using the Jacobian relations $\partial /\partial x=\ell_{x}/2\partial /\partial \xi$ and $\partial /\partial y=\ell_{y}/2\partial /\partial \eta$.

Applying the neutral equilibrium criterion $\delta^{2}\phi_{e}=0$ [53] leads to:

(22) $$\begin{array}{c} \delta^{2}\phi =\sum_{e=1}^{n_{e}}\delta^{2}\phi_{e}=\sum_{e=1}^{n_{e}}\left[\int_{\Omega_{e}}\left(\delta N^{⊤}\delta \varepsilon +\delta M^{⊤}\delta \kappa \right)d\Omega_{e}\right.\\ \left.+\int_{\Omega_{e}}\left(N^{⊤}\delta^{2}\varepsilon +M^{⊤}\delta^{2}\kappa \right)d\Omega_{e}\right]=0 \end{array}$$

The first integral of Equation (22) becomes the constitutive stiffness matrix of the element, calculated using the constitutive relations from classical laminated plate theory [46], where δN=Aδε+Bδκ and δM=Bδε+Dδκ. Note that the geometric non-linearity appears in the constitutive stiffness matrix due to $w_{,x}$, $w_{,y}$, and *v* in Equation (21). Therefore, the linear constitutive stiffness matrix of a finite element $K_{e}$ is calculated assuming $w_{,x},w_{,y},v=0$ in $B_{m}$ and $B_{b}$, leading to a 40×40 matrix defined by:

(23) $$K_{e}=\int_{x}\int_{y}\left(B_{m}^{⊤}AB_{m}+B_{m}^{⊤}BB_{b}+B_{b}^{⊤}BB_{m}+B_{b}^{⊤}DB_{b}\right)dxdy$$

The second integral of Equation (22) becomes the geometric stiffness matrix of the finite element ${K_{G}}_{0e}$, capturing the geometrically nonlinear effects of a linear pre-buckling membrane stresses $N_{0}={\left\{N_{0xx},N_{0yy},N_{0xy}\right\}}^{⊤}$ on the membrane stiffness. Noting that $\delta^{2}\kappa =0$ [24,53], the equation for ${K_{G}}_{0e}$ becomes:

(24) $$\begin{array}{c} {K_{G}}_{0e}=\underset{xy}{\;\int \int \;}\left[N_{0xx}S_{,x}^{w^{⊤}}S_{,x}^{w}+\right.\\ N_{0yy}\left(S_{,y}^{w^{⊤}}S_{,y}^{w}+\frac{1}{r^{2}}S^{v^{⊤}}S^{v}-\frac{1}{r}S^{v^{⊤}}S_{,y}^{w}-\frac{1}{r}S_{,y}^{w^{⊤}}S^{v}\right)\\ \left.N_{0xy}\left(S_{,y}^{w^{⊤}}S_{,x}^{w}+S_{,x}^{w^{⊤}}S_{,y}^{w}-\frac{1}{r}S^{v^{⊤}}S_{,x}^{w}-\frac{1}{r}S_{,x}^{w^{⊤}}S^{v}\right)\right]dxdy \end{array}$$

The contributions of all $n_{e}$ finite elements are added to build the global constitutive stiffness matrix K and the geometric stiffness matrix ${K_{G}}_{0}$ of the system:

(25) $$\begin{array}{c} K=\sum_{e=1}^{n_{e}}K_{e}\\ {K_{G}}_{0}=\sum_{e=1}^{n_{e}}{K_{G}}_{0e} \end{array}$$

The linear pre-buckling stress field of one finite element $N_{0xx},N_{0yy},N_{0xy}$ is calculated from the corresponding nodal displacements ${u_{0}}_{e}$ as:

(26) $$N_{0}=\left\{\begin{array}{c} N_{0xx}\\ N_{0yy}\\ N_{0xy} \end{array}\right\}=AB_{M}{u_{0}}_{e}$$

where ${u_{0}}_{e}$ is directly extracted from the full pre-buckling displacement vector $u_{0}$ that can be obtained from a linear static analysis, derived from the equilibrium of Equation (20):

(27) $$u_{0}=K^{-1}f_{0}$$

with $f_{0}$ representing any general pre-buckling force, here calculated based on a uniformly distributed axially compressive unit force $P_{unit}=1$. Assuming that there is a stress state $N=\lambda N_{0}$ that leads to the condition $\delta^{2}\phi =0$, the problem consists of finding the value of λ, such that:

(28) $$\delta u^{⊤}\left(K+\lambda {K_{G}}_{0}\right)\delta u=0$$

which holds true for any variation δu, such that the required condition for the equality of Equation (28) is:

(29) $$\det\left(K+\lambda {K_{G}}_{0}\right)=0$$

Equation (29) represents a linear buckling eigenvalue problem, solved numerically in the present study by means of the locally optimal block preconditioned conjugate gradient method [54], implemented in SciPy [55]. The lowest eigenvalue is the critical linear buckling load multiplier $\lambda_{c}$, from which the critical compressive load can be calculated with $P_{critical}=\lambda_{c}$.

The integration of $K_{e}$ and ${{K_{G}}_{0}}_{e}$ over the finite element domains are performed numerically using standard Gauss-quadrature with 4×4 integration points per element. The authors verified that this amount of integration points leads to a converged behavior even for variable-stiffness filament-wound cylinders [40]. For each integration point, the local shell constitutive properties are calculated using the smeared approach proposed by Castro et al. [36] based on a constant-volume constraint, verified against a discreet thickness modeling by Vertonghen and Castro [37].

## 4. Post-Buckling Stiffness

The post-buckling stiffness $b_{I}$, also known as $b_{factor}$, is herein calculated using the proposed SC-BFSC element, based on Koiter’s asymptotic approach. Given a total potential energy functional ϕ[u,λ] that depends on displacements u and a scalar load multiplier λ, a pre-buckling static equilibrium with solution $u_{0}$ that corresponds to a load level $\lambda_{0}$ can be described as per Equation (30), where the notation $\phi^{\prime }\delta u$ is used instead of δϕ to conveniently express the functional variation as a tensor product between the Fréchet derivative $\phi^{\prime }$ and the variation of the vector containing all degrees of freedom δu [24,25,56]. Assume that there exists at least one point of equilibrium that intersects [u(λ),λ] at a bifurcation point $\left[u_{c},\lambda_{c}\right]$, such that $u_{c}=\lambda_{c}u_{0}$, with $\lambda_{c}$ representing the critical buckling load, or critical bifurcation load, such that:

(30) $$\delta \phi \left[u_{0},\lambda_{0}\right]=\phi^{\prime }\left[u_{0},\lambda_{0}\right]\delta u=0$$

Koiter [11] proposed expressing $u-u_{c}$ and $\lambda -\lambda_{c}$ using asymptotic expansions to represent the difference between the current displacements and the displacements at the bifurcation point with the corresponding load increment $\lambda -\lambda_{c}$:

(31) $$\begin{array}{c} u-u_{c}=v=\xi u_{I}+\xi^{2}u_{\mathrm{II}}+\xi^{3}u_{\mathrm{III}}+\cdots \\ \lambda -\lambda_{c}=a_{I}\lambda_{c}\xi +b_{I}\lambda_{c}\xi^{2}+\cdots \end{array}$$

where:

(1) ξ is a scalar parameter;
(2) $u_{I}$ is a first-order field, taken directly from one or a linear combination of multiple linear buckling modes. Vector $u_{I}$ is customarily re-scaled by dividing with the maximum normal displacement amplitude and multiplying by the plate or shell thickness;
(3) $u_{\mathrm{II}}$ is a second-order field that provides a correction to the first-order field;
(4) the third-order field $u_{\mathrm{III}}$, and higher fields, are assumed to have a negligible contribution;
(5) $a_{I}$ and $b_{I}$ are, respectively, the first- and second-order load parameters to be determined.

Equation (31) is a reduced-order model (ROM) relating the load λ and displacement u around the equilibrium point $\left[u_{c},\lambda_{c}\right]$.

Note that Equation (31) consists of a reduced-order model to calculate displacements u based on a pre-buckled state $u_{c}$ with known first- and second-order fields $u_{I}$ and $u_{\mathrm{II}}$. The coefficient ξ can be determined for each known load level λ, after the calculation of the coefficients $a_{I}$ and $b_{I}$.

The expression given by Equation (31) can be applied to the expanded total potential energy functional of Equation (32) [24,25], leading to:

(32) $$\begin{array}{ll} \phi^{\prime }\left[u,\lambda \right]\delta u & =\left(\phi_{c}^{\prime \prime }+{\overset{•}{\phi }}_{c}^{\prime \prime }\left(\lambda -\lambda_{c}\right)+\frac{1}{2}{\overset{••}{\phi }}_{c}^{\prime \prime }{\left(\lambda -\lambda_{c}\right)}^{2}+\cdots \right)v\delta u\\ & +\frac{1}{2}\left(\phi_{c}^{\prime \prime \prime }+{\overset{•}{\phi }}_{c}^{\prime \prime \prime }\left(\lambda -\lambda_{c}\right)+\frac{1}{2}{\overset{••}{\phi }}_{c}^{\prime \prime \prime }{\left(\lambda -\lambda_{c}\right)}^{2}+\cdots \right)v^{2}\delta u\\ & +\frac{1}{6}\left(\phi_{c}^{iv}+{\overset{•}{\phi }}_{c}^{iv}\left(\lambda -\lambda_{c}\right)+\frac{1}{2}{\overset{••}{\phi }}_{c}^{iv}{\left(\lambda -\lambda_{c}\right)}^{2}+\cdots \right)v^{3}\delta u\\ +\cdots & \end{array}$$

The resulting formula is shown in Equation (33), where the terms multiplying $\xi^{2}$ and $\xi^{3}$ are collected. Note that this expansion concerns only a single mode, and the reader is referred to Castro and Jansen [24,25] for details about the multi-modal expansion adopting the same notation. It follows that

(33) $$\begin{array}{ccc} \xi^{2}\left[2a_{I}\lambda_{I}u_{I}{\overset{•}{\phi }}_{c}^{\prime \prime }+\phi_{c}^{\prime \prime \prime }u_{I}u_{I}+2\phi_{c}^{\prime \prime }u_{\mathrm{II}}\right]\delta u & \; & \;\\ +\xi^{3}[6\lambda {\overset{•}{\phi }}_{c}^{\prime \prime }u_{I}b_{I}+\phi_{c}^{iv}u_{I}u_{I}u_{I}+6\phi_{c}^{\prime \prime \prime }u_{I}u_{\mathrm{II}} & \; & \;\\ +12{\overset{••}{\phi }}_{c}^{\prime \prime }\lambda^{2}u_{I}a_{I}+12{\overset{•}{\phi }}_{c}^{\prime \prime \prime }\lambda_{i}a_{I}u_{I}u_{I}]\delta u+\cdots & = & 0 \end{array}$$

where $\phi_{c}^{\prime \prime }$ is the tangent stiffness matrix; the calculation of ${\overset{•}{\phi }}_{c}^{\prime \prime }$, ${\overset{•}{\phi }}_{c}^{\prime \prime \prime }$, ${\overset{••}{\phi }}_{c}^{\prime \prime }$, $\phi_{c}^{\prime \prime \prime }$, and $\phi_{c}^{iv}$ is discussed in detail by Castro and Jansen [24,25]. For the expanded equilibrium to be stationary, each term in Equation (33) must vanish separately. Assuming $\delta u=u_{I}$ in Equation (33), the expressions for $a_{I}$ and $b_{I}$ of Equation (31) can be obtained, as given, respectively, in Equations (34) and (35):

(34) $$a_{I}=-\frac{1}{2\lambda_{i}}\frac{\phi_{c}^{\prime \prime \prime }u_{I}u_{I}u_{I}}{{\overset{•}{\phi }}_{c}^{\prime \prime }u_{I}u_{I}}$$

(35) $$\begin{array}{c} b_{I}=\frac{-1}{6\lambda {\overset{•}{\phi }}_{c}^{\prime \prime }u_{I}u_{I}}[\phi_{c}^{iv}u_{I}u_{I}u_{I}u_{I}+6\phi_{c}^{\prime \prime \prime }u_{I}u_{I}u_{\mathrm{II}}\\ +3\lambda {\overset{•}{\phi }}_{c}^{\prime \prime \prime }a_{I}u_{I}u_{I}u_{I}+3\lambda^{2}{\overset{••}{\phi }}_{c}^{\prime \prime }a_{I}^{2}u_{I}u_{I}] \end{array}$$

The second-order fields $u_{\mathrm{II}}$ needed for the calculation of $b_{I}$ can be obtained by first calculating a non-orthogonal second-order field ${\bar{u}}_{\mathrm{II}}$. The calculation of ${\bar{u}}_{\mathrm{II}}$ can be conducted directly from Equation (33), knowing that the term multiplying $\xi^{2}$ must vanish separately:

(36) $${\bar{u}}_{\mathrm{II}}={\left[\phi_{c}^{\prime \prime }\right]}^{-1}\left(-\frac{1}{2}\phi_{c}^{\prime \prime \prime }u_{I}u_{I}-a_{I}\lambda u_{I}{\overset{•}{\phi }}_{c}^{\prime \prime }\right)$$

The orthogonal second-order field vector $u_{\mathrm{II}}$ in the single-modal asymptotic expansion can be obtained after a Gram–Schmidt orthogonalization [57] operation, used to remove the components of ${\bar{u}}_{\mathrm{II}}$ that are parallel to the linear buckling mode used in the single-modal expansion named $u_{I}$, as formulated in Equation (37).

(37) $$u_{\mathrm{II}}={\bar{u}}_{\mathrm{II}}-u_{I}\frac{\langle {\bar{u}}_{\mathrm{II}},u_{I}\rangle }{\langle u_{I},u_{I}\rangle }$$

This topic concludes the discussion of the modeling strategy, with the exposition that follows focusing on the application and performance evaluation of three machine learning (ML) strategies applied on the optimization problem of Equation (11).

## 5. Machine Learning Strategies for Meta-Modeling

An interesting characteristic of machine learning algorithms is their ability to generalize the training experience and provide an unexpected output that best fulfills the objective function, predicting future events or scenarios that are not explicitly mapped in the training process, and that can, therefore, reach results that are unexpected and non-intuitive. Among a myriad of alternatives for implementing a learning strategy, a comprehensive overview of popular strategies and resources is presented by Russell [58].

In the present work, the viability of the Support Vector Machine (Section 5.2), the Kriging surrogate (Section 5.3), and the Random Forest (Section 5.4) algorithms are investigated as learning strategies to meta-model and optimize cylinder design. Numerical calculations were performed by several packages of the R software platform [59]. The discussion starts with the design of the experiments (Section 5.1).

### 5.1. Design of Experiment

The steering radius $r_{CTS}$, the number *n* of $c_{2}$ regions (Equation (6)), the ratio ${c_{2}}_{ratio}$ (Equation (4)), the tow angle $\theta_{1}$ at region 1, and the tow angle $\theta_{2}$ at region 2 constitute the five design variables

(38) $$v=[r_{CTS},n,{c_{2}}_{ratio},\theta_{1},\theta_{2}]$$

where $v_{1}=\left\{r_{CTS}\in R\;|\;0.05\leq r_{CTS}\leq 0.20\right\},v_{2}=\left\{n\in N\;|\;1\leq n\leq 12\right\},v_{3}=\left\{{c_{2}}_{ratio}\in R\;|\;0\leq {c_{2}}_{ratio}\leq 1\right\},v_{4}=\left\{\theta_{1}\in R\;|\;0\leq \theta_{1}\leq 75\right\},$ and $v_{5}=\left\{\theta_{2}\in R\;|\;0\leq \theta_{2}\leq 75\right\}$. The real-valued variables $v_{4}$ and $v_{5}$ are limited to two decimal places, in order to be closer to a viable solution for manufacturing.

The response in terms of buckling load $P_{critical}\left(v\right)$ and post-buckling stiffness $b_{I}\left(v\right)$ are evaluated throughout the design space by means of a Design of Experiment (DOE) where a set of 2000 feasible points is chosen from the design envelope

(39) $$\begin{array}{c} 0.05\leq r_{CTS}\leq 0.20\\ 1\leq n\leq 12\\ 0\leq {c_{2}}_{ratio}\leq 1\\ 0\leq \theta_{1}\leq 75\\ 0\leq \theta_{2}\leq 75 \end{array}$$

by means of a Latin hypercube sampling methodology, as available in the LHS toolbox [60]. The DOE is publicly available in a dataset published by the authors [61].

**Figure 3:**
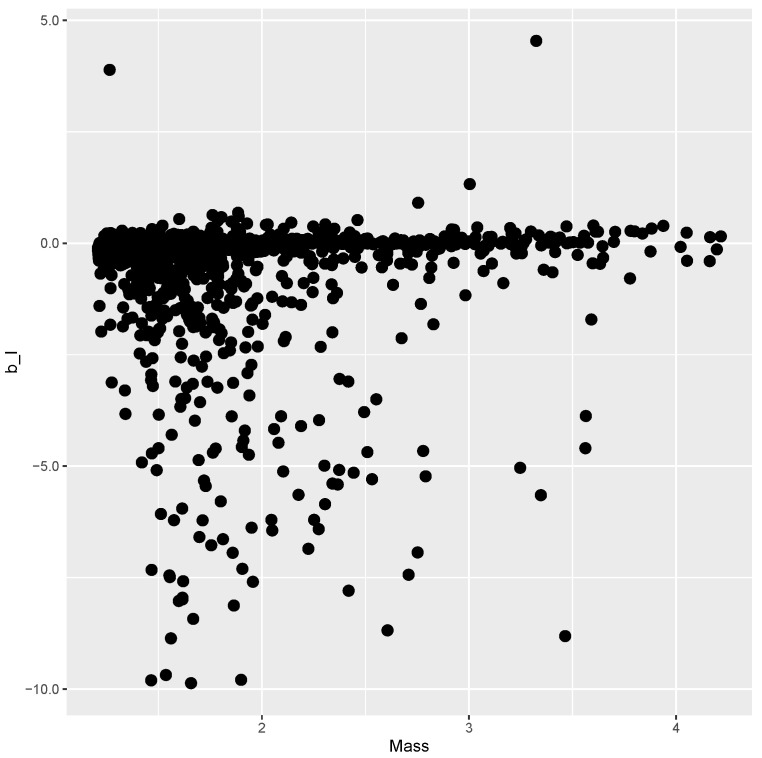
Mass and $b_{I}$ of sample points.

**Figure 4:**
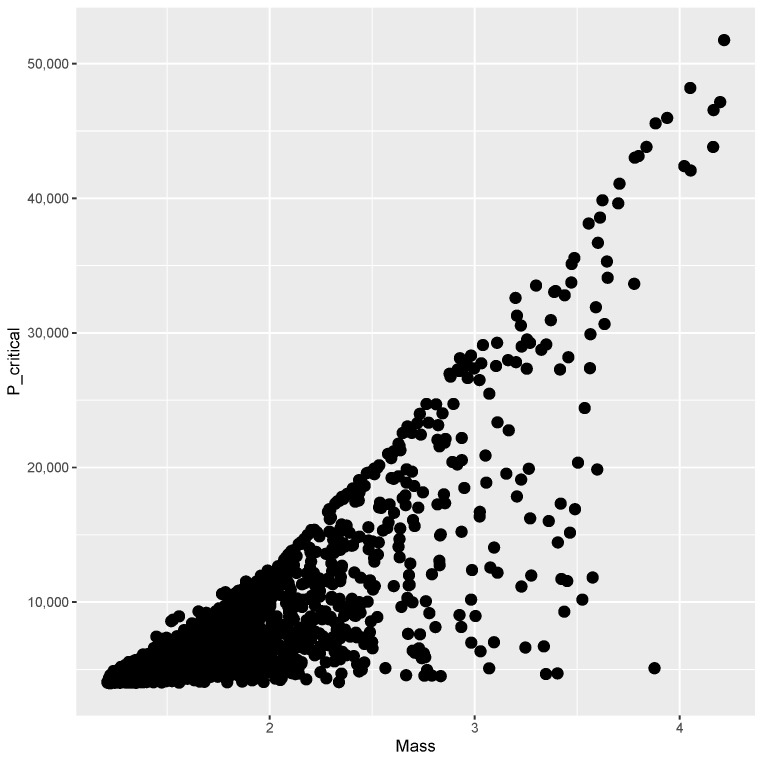
Mass and $P_{critical}$ of sample points.

The finite element model is evaluated at each point of the DOE, and the results are presented below. Figure 3 shows the mass versus $b_{I}$ obtained from every evaluation within the DOE, where no clear correlation between the variables can be seen. Figure 4 plots mass versus $P_{critical}$, showing a Pareto frontier at the upper limit of $P_{critical}$ for increasing values of mass.

The effectiveness of meta-modeling the $b_{I}$, mass, and $P_{critical}$ indices by means of SVM, Kriging, and Random Forest methodologies are discussed in Section 5.2, Section 5.3 and Section 5.4, respectively. The numerical results, obtained from a set of $n_{sample}=2000$ feasible points, are divided into two groups: the first group of $n_{training}=1600$ points is used in the training phase, to adjust the models, and the second group of $n_{test}=400$ points is used in the test phase, to infer the quality of the models.

**Figure 5:**
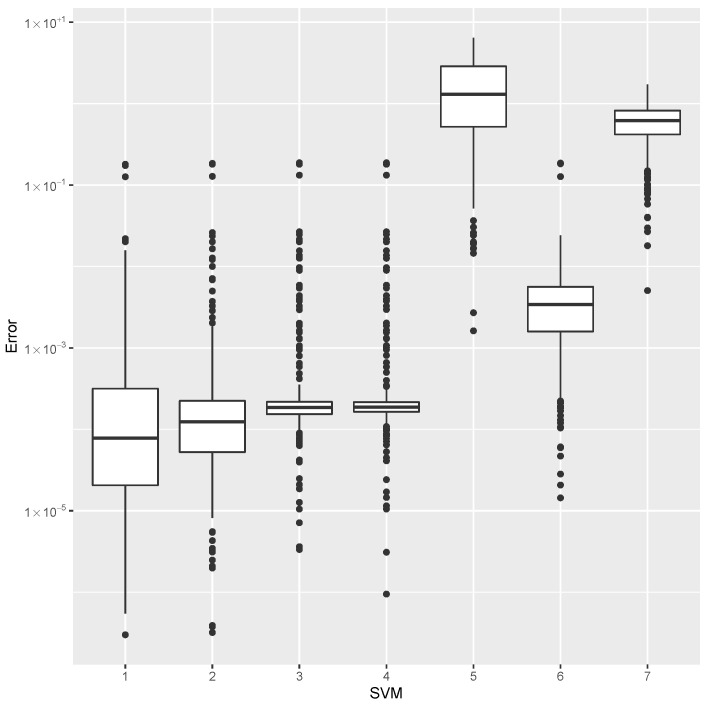
Error dispersion of the $b_{I}$ estimate using distinct SVM kernel options.

**Figure 6:**
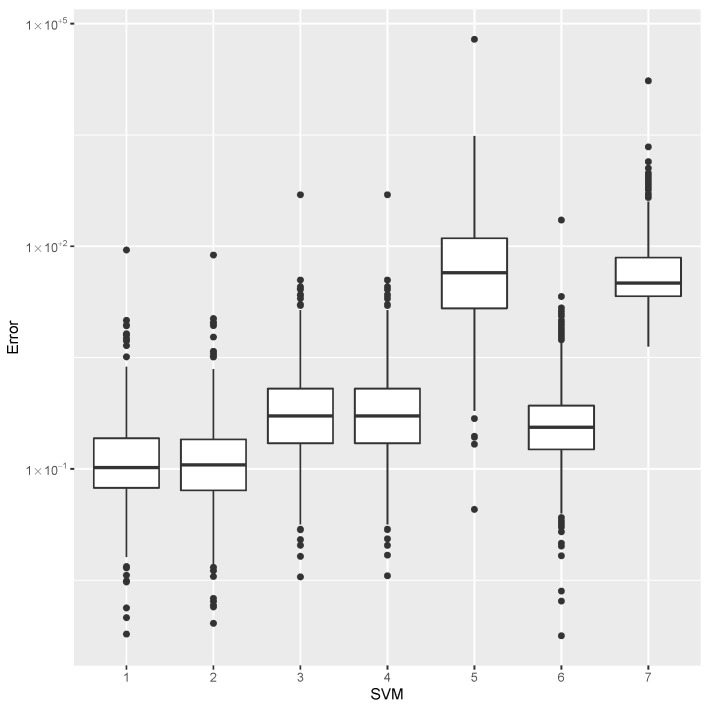
Error dispersion of the mass estimate using distinct SVM kernel options.

**Figure 7:**
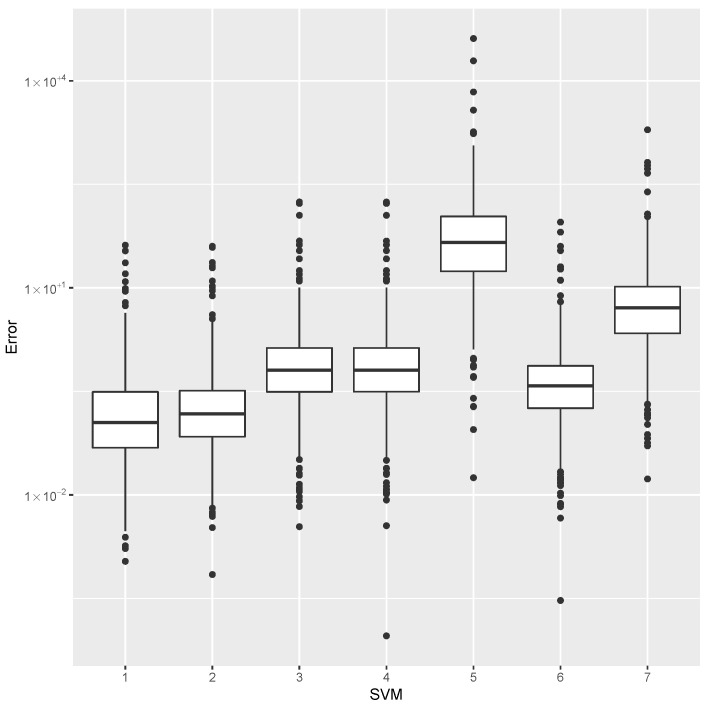
Error dispersion of the $P_{critical}$ estimate using distinct SVM kernel options.

**Figure 8:**
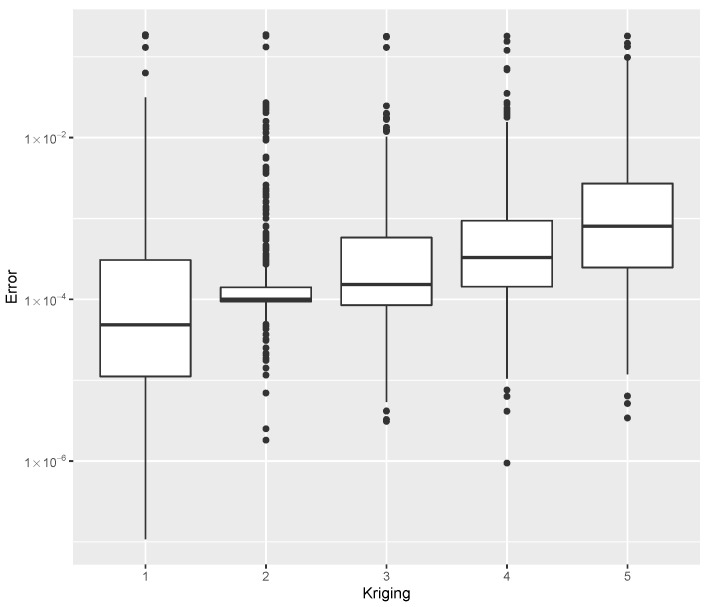
Error dispersion of the $b_{I}$ estimate using distinct Kriging kernel options.

**Figure 9:**
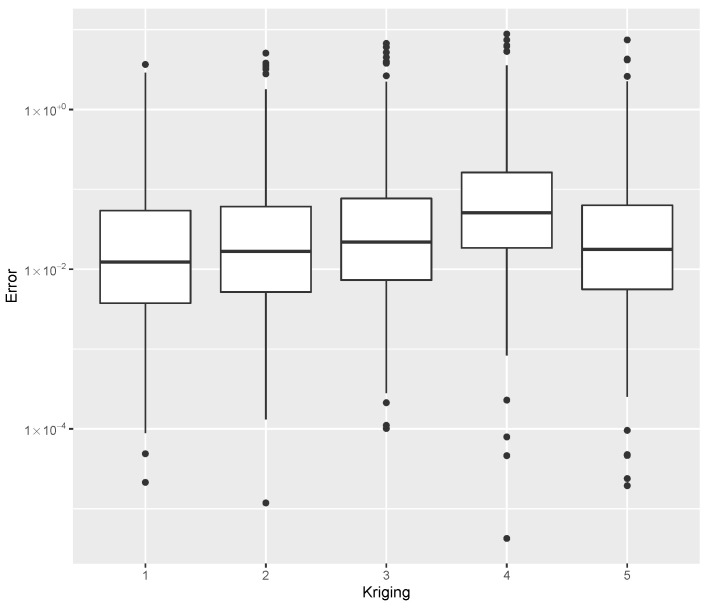
Error dispersion of the mass estimate using distinct Kriging kernel options.

**Figure 10:**
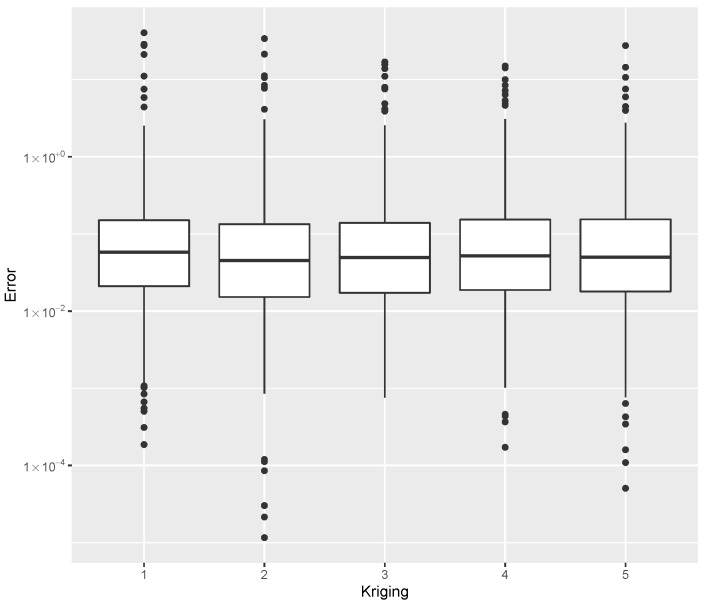
Error dispersion of the $P_{critical}$ estimate using distinct Kriging kernel options.

**Figure 11:**
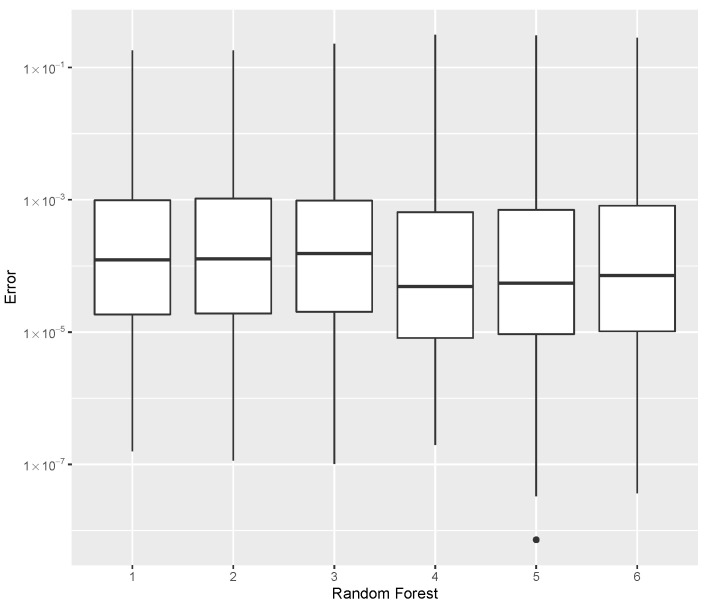
Error dispersion of the $b_{I}$ estimate using distinct Random Forest parameters.

**Figure 12:**
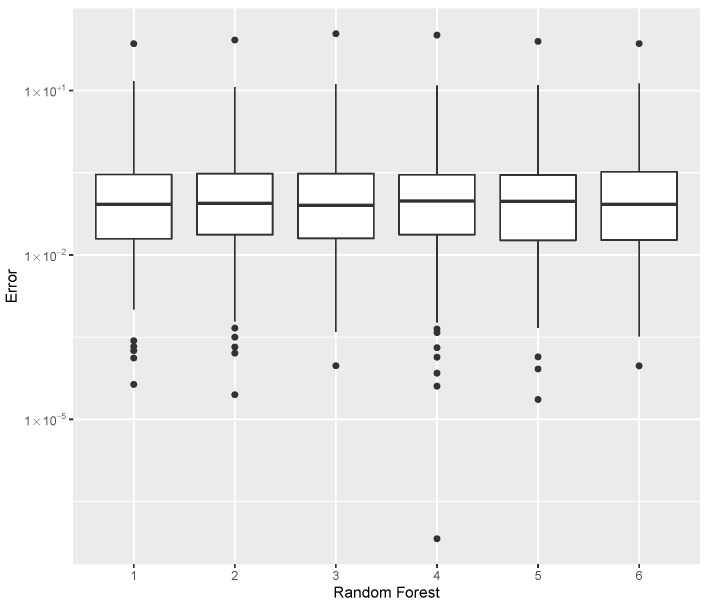
Error dispersion of the mass estimate using distinct Random Forest parameters.

**Figure 13:**
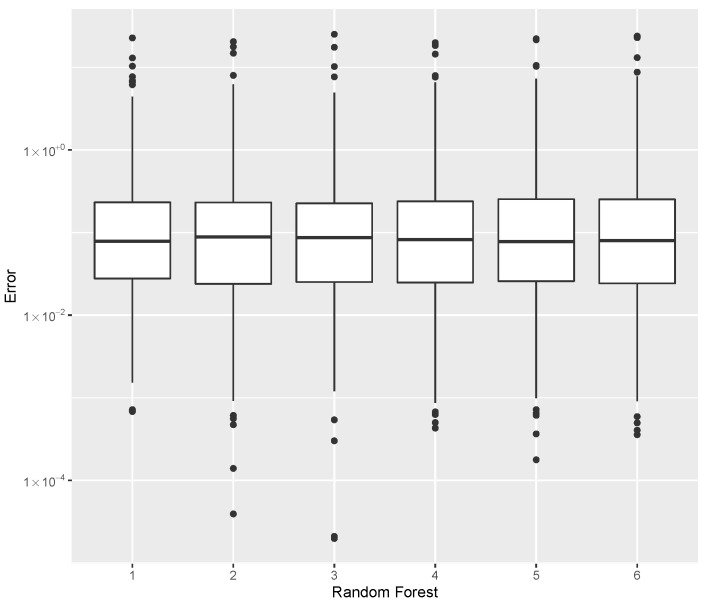
Error dispersion of the $P_{critical}$ estimate using distinct Random Forest parameters.

**Figure 14:**
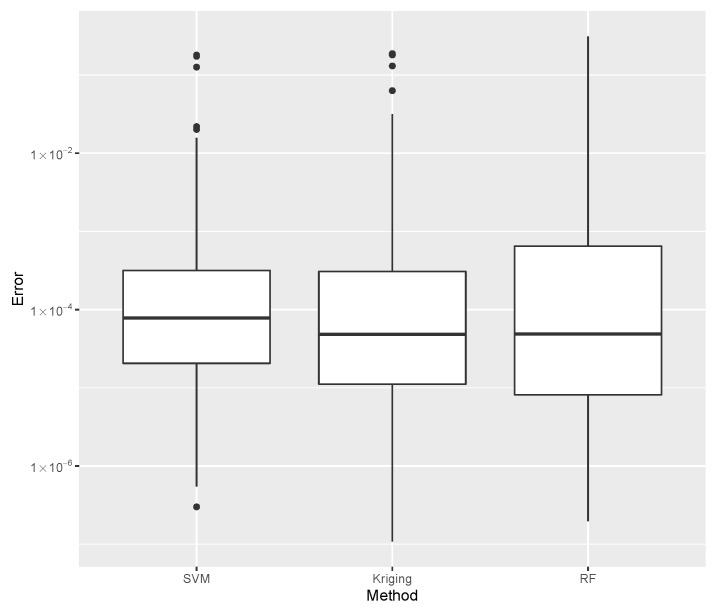
Comparison of $b_{I}$ error dispersion considering the best SVM, Kriging, and Random Forest estimates.

**Figure 15:**
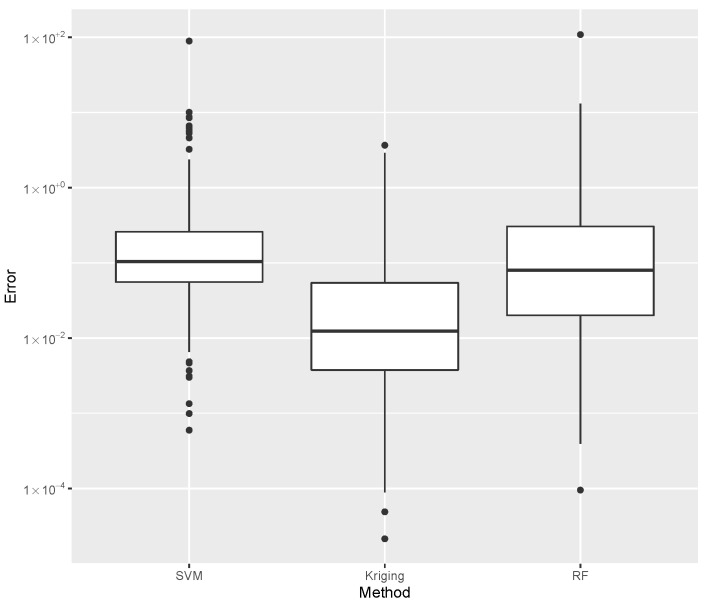
Comparison of mass error dispersion considering the best SVM, Kriging, and Random Forest estimates.

**Figure 16:**
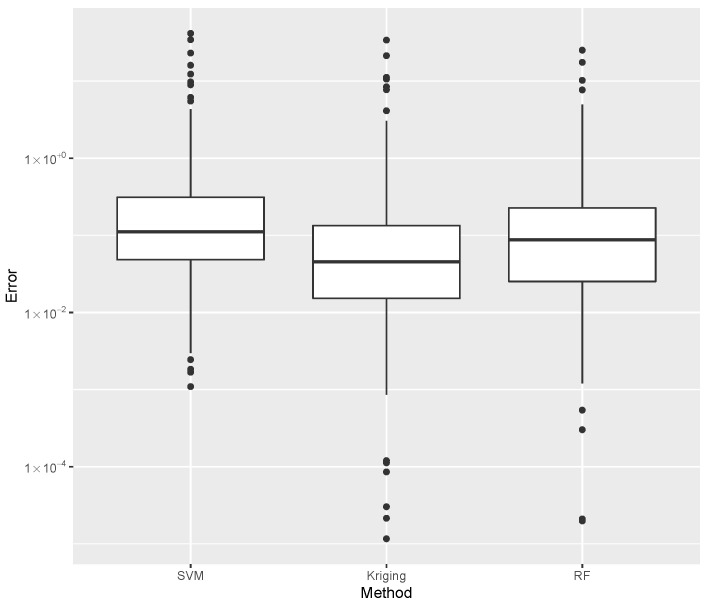
Comparison of $P_{critical}$ error dispersion considering the best SVM, Kriging, and Random Forest estimates.

The relative error E(v) of the meta-model is used to quantify the deviance between the actual f(v) and estimated $\hat{f}\left(v\right)$ values of a design v, and is evaluated as

(40) $$E\left(v\right)=\frac{|\hat{f}\left(v\right)-f\left(v\right)|}{\left|f(v)\right|}$$

while providing estimates for the post-buckling stiffness $b_{I}$, the mass, and the buckling load $P_{critical}$. There are several boxplot diagrams presented in Figure 5, Figure 6, Figure 7, Figure 8, Figure 9, Figure 10, Figure 11, Figure 12, Figure 13, Figure 14, Figure 15 and Figure 16, whose data are calculated following Equation (40). The results summarized in Table 1, Table 2, Table 3, Table 4, Table 5, Table 6, Table 7, Table 8 and Table 9 are also obtained by using Equation (40). As the sample points are randomly chosen in the design space, from the project perspective, it is important to identify the points along the frontier that offer a positive post-buckling stiffness, herein represented by the criteria $b_{I}>0$. This discussion will be expanded in Section 6.

### 5.2. Support Vector Machine

The standard SVM is formulated as a classifier whose decision function is represented by a hyper-plane that maximizes the distance of separated samples from different classes. The methodology ultimately finds a linear combination of features that characterizes or separates two or more classes of objects or events (as described by $y_{i}$). Since the convex optimization problem is solved by a deterministic algorithm, for a specific input dataset, the same optimal hyper-plane parameter is obtained, as a solution of Equations (41)–(43).

Formally, the minimization problem is given by

(41) $$\min_{w,\xi ,b}\left({0.5\|w\|}^{2}+C\sum_{i=1}^{n_{training}}\xi_{i}\right)$$

subject to

(42) $$y_{i}\left(\langle \phi \left(v_{i}\right),w\rangle +b\right)\geq 1-\xi_{i},\;\forall i=1,\ldots ,m$$

where

(43) $$\xi_{i}\geq 0,\;\forall i=1,\ldots ,m$$

and $m\leq n_{training}$ is the number of active constraints. A detailed discussion about the SVM method and variants can be found in Awad and Khanna [62] and Salcedo-Sanz and Rojo-Álvarez [63].

In the present study, the SVM methodology is evaluated considering the characteristics of seven different kernels (as expressed by ϕ in Equation (42)): Laplace, RBF, Polynomial, Vanilla, Hyperbolic Tangent, Bessel, and ANOVA. The executions are performed thought the Kernlab toolbox [64]. A set of 2000 feasible points is chosen from the design envelope using a Latin hypercube sampling methodology that is available in the LHS toolbox [60]. Then, the set is divided into two groups: the first group of 1600 points is used in the training phase, to adjust the models, and the second group of 400 points is used in the test phase, to infer the quality of the models. As a result, the performance of each kernel will be evaluated against a set of 400 designs not used in the training phase.

The minimum error, the median error, and the maximum error in the estimation of the $b_{I}$ are shown in Table 1. The error dispersions (Equation (40)) of all SVM methods are compared in Figure 5.

The median of the error dispersion is used as a metric to identify the best adjustment. According to this criteria, the first model, using the Laplace kernel, is found to be the best $b_{I}$ predictor.

For the mass estimation, the minimum error, the median error, and the maximum error are shown in Table 2. The error dispersion of the mass estimates using all SVM methods are compared in Figure 6. Based on the median error, the Laplace kernel is the best $b_{I}$ predictor.

For the estimation of $P_{critical}$, the minimum error, the median error, and the maximum error are shown in Table 3. The error dispersion of the $P_{critical}$ estimate is shown in Figure 7. The Laplace kernel is the best $P_{critical}$ predictor, based on the median error.

According to the metric of the median error, the SVM combined with the Laplace kernel is the best predictor for the design space created under the proposed parameterization of variable-stiffness cylinders.

### 5.3. Kriging Surrogate

Haeri and Fadaee [65] present a reliability analysis of laminated composites using a Kriging surrogate model, where the performance is compared with results obtained from a radial basis neural network. The comparison results confirm the computational cost efficiency and accuracy of Kriging model for reliability analyses of laminated composites. The Kriging method [66,67] treats the function of interest as a realization of a stochastic process y(v) by means of the equation:

(44) $$y\left(v\right)=\sum_{j=1}^{k}\beta_{j}f_{j}\left(v\right)+Z\left(v\right)$$

where f(v) is a linear regression of the data, with *k* regressors modeling the drift of the process mean over the domain. The second part, Z(v), is a model of a Gaussian and stationary random process with zero mean and covariance

(45) $$\mathrm{cov}\left(v_{1},v_{2}\right)=\sigma^{2}R\left(v_{1},v_{2}\right)$$

between two observations, $v_{1}$ and $v_{2}$. The authors of [68] present an overview of common spatial correlation functions $R(v_{1},v_{2})$ for approximating a deterministic computer model and the influence of parameter selection on the properties of these functions.

In the present study, the Kriging methodology is evaluated considering the characteristics of five different spatial correlation kernels: Gaussian, Matern $5_{2}$, Matern $3_{2}$, Exponential, and Power-Exponential. The performance of each model is evaluated against a set of 400 testing designs not used in the training phase. The executions are performed through the DiceKriging toolbox [69].

A set of 2000 feasible points is chosen from the design envelope using a Latin hypercube sampling methodology, available in the LHS toolbox [60]. Then, the set is divided into two groups: the first group of 1600 points is used in the training phase, to adjust the models, and the second group of 400 points is used in the test phase, to infer the quality of the models.

For the $b_{I}$ estimation using Kriging with different kernels, the minimum error, the median error, and the maximum error as evaluated by Equation (40) are shown in Table 4. The error dispersion of all Kriging methods are compared in Figure 8. The median of the error dispersion is used as a metric to identify the best adjustment. According to this criteria, the first model, using the Gaussian kernel, is found to be the best $b_{I}$ predictor.

The minimum error, the median error, and the maximum error in the estimation of the mass *m* are shown in Table 5. The error dispersion of the mass estimates using all Kriging methods are compared in Figure 9, where the first model, based on the Gauss kernel, is the best mass predictor according to the metric of the median error.

For the estimation of $P_{critical}$, the errors are shown in Table 6. The error dispersion of the $P_{critical}$ estimate is shown in Figure 10. The Matern $3_{2}$ kernel is the best $P_{critical}$ predictor, based on the median error; the other kernels lead to similar results, and therefore, they can be chosen without compromising the analysis.

It noteworthy that there is no single option with superior performance on all three datasets (mass, $b_{I}$, and $P_{critical}$). In this case, the performance is dependent on the data under consideration.

### 5.4. Random Forest

The Random Forest [70] algorithm is a supervised learning procedure which operates according to the divide-and-conquer principle: sample fractions of the data, grow a randomized tree predictor on each small piece, and then aggregate these predictors together. It can be applied to a wide range of prediction problems and have few parameters to tune.

A Random Forest consists in a predictor composed by a collection of *M* randomized regression trees. For the *j*-th tree in the family, the predicted value at the query point *x* is denoted by $m_{n}\left(x;\Theta_{j},D_{n}\right)$, where $\Theta_{1},\ldots ,\Theta_{M}$ are independent random variables.

A key result of such methodology is that as the number of trees increases, all sequences of generalization error converge almost surely. This result explains why Random Forests do not overfit as more trees are added, but produce a limiting value of the generalization error.

In the present study, the Random Forest is evaluated considering the number of trees to grow (tree ∈{50,100,200}) and the number of variables randomly sampled as candidates at each split (split ∈{3,5}). The performance of each model is evaluated against a set of $n_{test}=400$ designs not used in the training phase. The executions are performed thought the randomForest toolbox in R [71].

The minimum error, the median error, and the maximum error in the estimation of the $b_{I}$, as evaluated by Equation (40), are shown in Table 7. The error dispersion of all Random Forest methods are compared in Figure 11. The median of the error dispersion is used as a metric to identify the best adjustment. According to this criteria, the fifth model, using the 100 trees and 5 splits, is found to be the best $b_{I}$ predictor.

The errors in the estimation of the mass are shown in Table 8, while the dispersion using all Random Forest methods are compared in Figure 12. The parameters tree = 200 and split = 5 provided the best mass predictor, based on the median error.

For the estimation of $P_{critical}$, the error data is given in Table 9, with the error dispersion shown in Figure 13 where, based on the median error, the parameters tree = 200 and split = 3 provided the best $P_{critical}$ predictor.

Different values for trees and splits resulted in small performance deviations. A similar profile is observed in all the datasets considered. As a result, this method is seen as a robust choice for meta-modeling which does not require specific knowledge about parameter tuning in order to obtain good results.

## 6. The Inverse Problem

Raissi et al. [72] proposed a physics-informed neural network for solving inverse problems after learning the unknown model parameters, by means of gradient-based learning, of the Navier–Stokes and Korteweg–de Vries equations. Haghighat et al. [73] applied a similar approach to solid mechanic problems. Yan et al. [74] applied extreme machine learning to the analysis of composite structures, demonstrating how the framework can be applied to inverse problems with the aim of finding optimal designs.

A typical challenge that occurs in the design phase is the ability to satisfy a trade-off between mass and stiffness. In order to meet the changing need for various requirements, the possibility of exploring the design space and the optimal compromise between variables are important aspects that are discussed now.

In the present study, the direct problem consists in calculating the mass *m*, the critical buckling load $P_{critical}$, and the post-buckling stiffness $b_{I}$ from the input design variables $v_{1},\cdots ,v_{5}$ (Equation (38)). The inverse problem to be solved with the proposed machine learning framework consists of finding the values of the design variables $v=[v_{1},\cdots ,v_{5}]$ for a given *m*, $P_{critical}$, and $b_{I}$. The solution of the inverse problem is computed as outlined in Algorithm 3 below, where the relative error between the index estimate and the actual index value is computed through Equation (40). The model with the smallest median of error values is chosen as representative of each category: SVM, Kriging, and Random Forest.

While evaluating $b_{I}$, the error dispersion of the selected methods are compared, as shown in Figure 14. Note that the Random Forest presented the lowest error median; thus, it is used in the computations of the inverse problem discussed later. For the mass evaluation, the error dispersion of the selected methods are compared, as is shown in Figure 15. The Kriging algorithm is used in the computations discussed next because it presented the lowest median error.

For the evaluation of $P_{critical}$, the error dispersion of the selected methods are compared, as is shown in Figure 16. The Kriging algorithm was selected as the best meta-modeling technique to compute $P_{critical}$, although the best results of each of the three groups presented similar performances. In this case, the Random Forest or the SVM are used without compromising the result, and both methods show comparable computational costs.

The steps performed in adjusting the models for a given dataset—the training phase—are shown in Algorithm 2.

Given a set of target values $\tilde{b},\tilde{m}$, and $\tilde{p}$, representing the desired $b_{I}$, mass, and $P_{critical}$, the inverse problem is computed by means of the minimization of

(46) $$\begin{array}{ccc} H(\tilde{b},\tilde{m},\tilde{p},v_{j}) & = & \alpha_{1}{\left(F_{b}-\tilde{b}\right)}^{2}+\alpha_{2}{\left(F_{m}-\tilde{m}\right)}^{2}+\alpha_{3}{\left(F_{p}-\tilde{p}\right)}^{2}\\ \; & \; & +\alpha_{4}\left[\sum_{j=1}^{5}{\left(G_{j}-v_{j}\right)}^{2}\right] \end{array}$$

subject to

(47) $$\begin{array}{c} 0.05\leq v_{1}\leq 0.20\\ 1\leq v_{2}\leq 12\\ 0\leq v_{3}\leq 1\\ 0\leq v_{4}\leq 75\\ 0\leq v_{5}\leq 75 \end{array}$$

where $\alpha_{1},\;\alpha_{2},\;\alpha_{3}$, and $\alpha_{4}$ are weighting parameters, indicating preference in meeting each goal. Although the design values $v_{j}$ that solve the inverse problem are not known at the beginning of the optimization process, an useful estimate is provided by the meta-model $G_{j}$.
**Algorithm 2:** The training phase
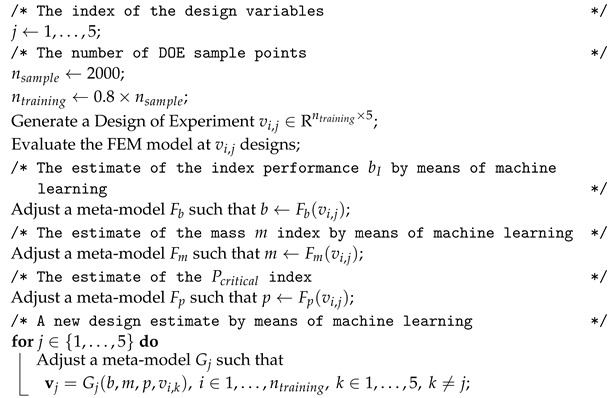


It is worth mentioning that Equation (46) retains the concepts provided by Equation (11), and extends the analysis by considering further information provided by $G_{j}$. Furthermore, the inequalities explicitly described by Equation (10) are implicit in the learning process of the $F_{b},F_{m},F_{p}$, and $G_{j}$ meta-models. The remaining box constraints (Equation (12)) will also be checked by Algorithm 3. As a result, the proposition of Equations (11) and (12) also constitutes a contribution of the present study.

After the training phase is accomplished (Algorithm 2), the computation of a new design $v_{j}^{*},\;j=1,\cdots ,5$ that solves the inverse problem is given by Algorithm 3. The effectiveness of this methodology in providing feasible designs is discussed in the following.
**Algorithm 3:** Obtaining the design as an inverse problem
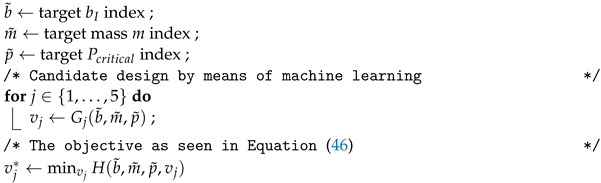


**Figure 17:**
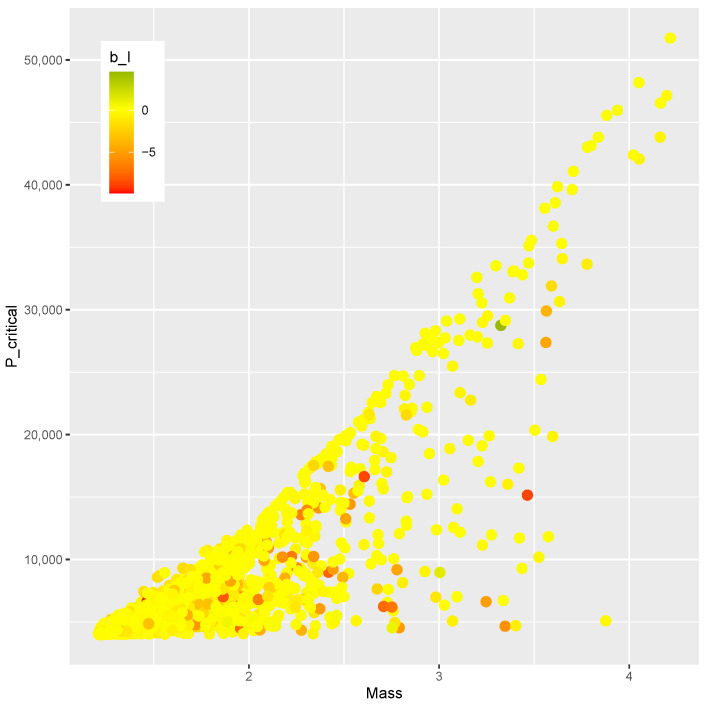
Mass *m*, $P_{critical}$, and $b_{I}$ of the DOE.

A Design of Experiments of $n_{sample}=2000$ points is generated by means of a Latin hypercube sampling algorithm [60]. The optimization phase is driven by a differential evolution strategy implemented in the DEoptim toolbox [75,76]. The mass *m*, the post-buckling stiffness $b_{I}$, and the buckling load $P_{critical}$ of each point are shown in Figure 17, where a correlation between the upper value of $P_{critical}$ and the mass can be noticed. However, there is no apparent correlation between mass and $b_{I}$, nor is there one between $P_{critical}$ and $b_{I}$. As a result, it would be of practical interest for the design optimization of the present study to identify designs on this Pareto-like frontier between *m* and $P_{critical}$ that contains a positive $b_{I}$.

**Figure 18:**
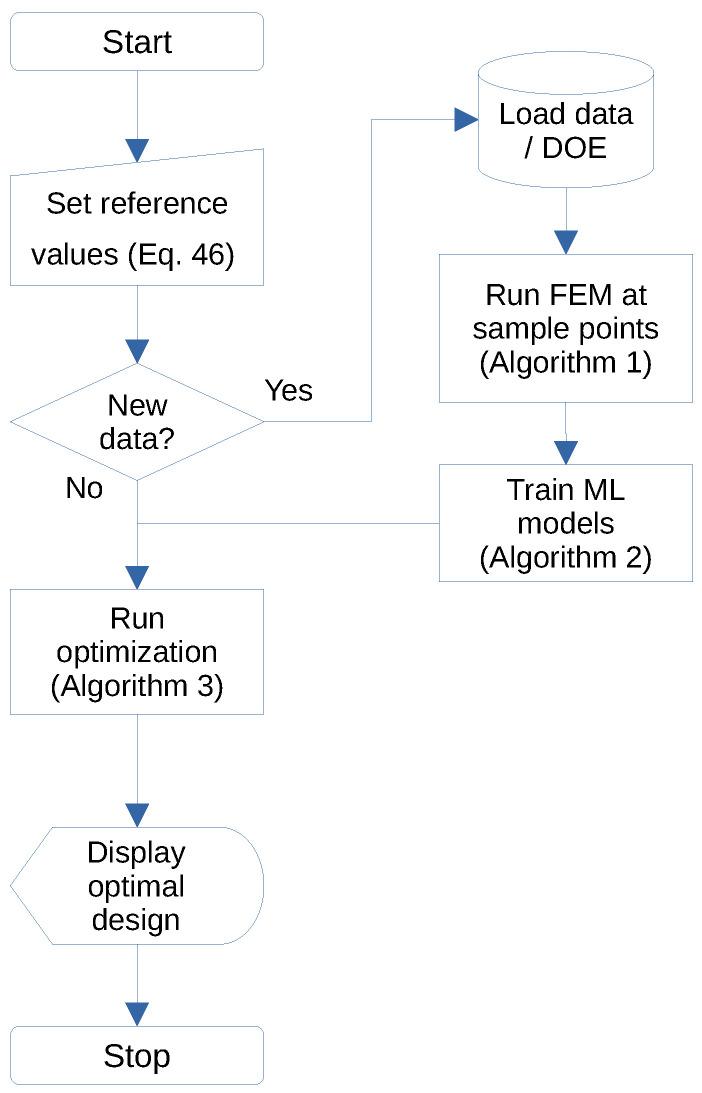
Workflow for obtaining the optimal design.

Using no further information about feasible designs, an arbitrary set of experiments are evaluated by this inverse problem algorithm. The entire process of modeling (Algorithm 1), training (Algorithm 2), and optimization (Algorithm 3) is summarized in Figure 18.

**Figure 19:**
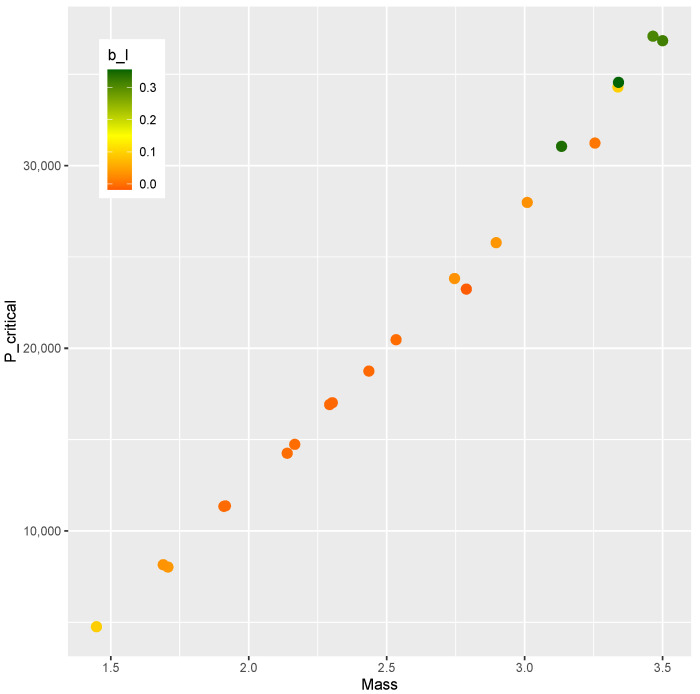
Mass *m* and $P_{critical}$ at critical designs.

The designs obtained as a response to the inverse problem are evaluated by the finite element model, resulting in the values of *m* and $P_{critical}$ shown in Figure 19. Those results resemble the trend found in the DOE when mass and $P_{critical}$ are compared. Therefore, the meta-models herein applied have proven effective in evaluating new designs in a region of interest.

**Figure 20:**
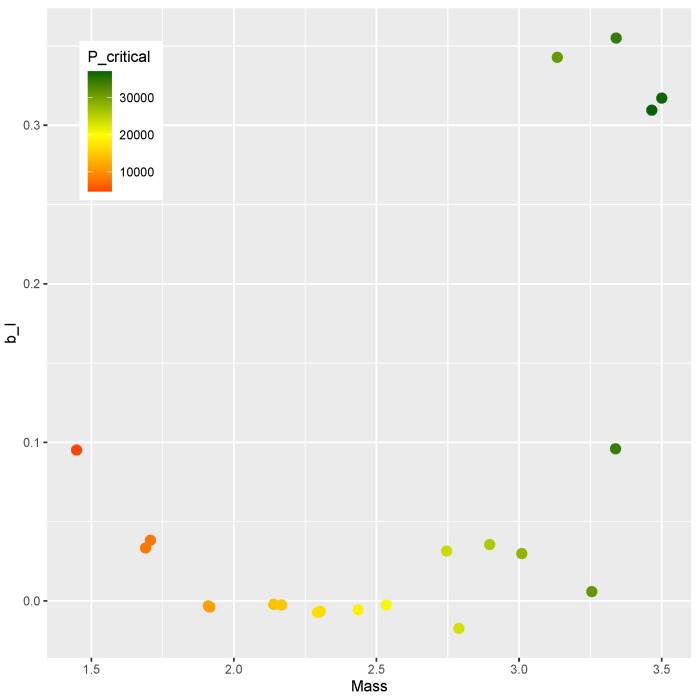
Mass *m* and $b_{I}$ at critical designs.

**Figure 21:**
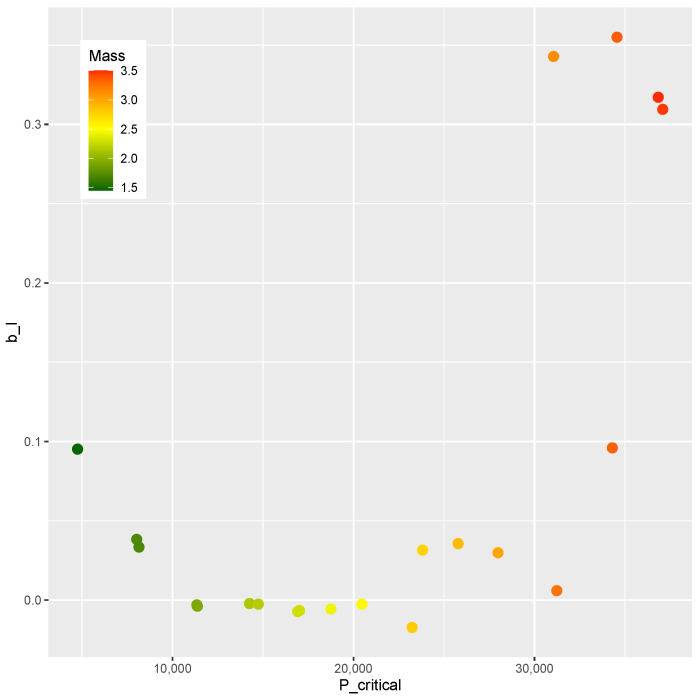
$P_{critical}$ and $b_{I}$ at critical designs.

The comparison between the mass and the post-buckling stiffness $b_{I}$ is presented in Figure 20. The feasible designs contain a positive $b_{I}$. The methodology was effective in identifying feasible designs in two ranges of mass (m<2 kg and m>2.5 kg). The comparison of $b_{I}$ and $P_{critical}$ is shown in Figure 21. Feasible designs ($b_{I}>0$) are found in the ranges $P_{critical}<$ 10,000 and $P_{critical}>$ 25,000.

A similar trend is found in the comparison of Figure 20 and Figure 21. Since the mass *m* and buckling load $P_{critical}$ are chosen in a correlated fashion, this similarity was expected. The design variables and the corresponding $b_{I}$, *m*, and $P_{critical}$ are presented in Table 10.

Two decimal digits are considered when calculating the values of the design variables $v_{4}$ and $v_{5}$ throughout the optimization process. This constraint addresses a manufacturing requirement. Moreover, the $v_{2}$ design variable has integral values. The corresponding results are shown in columns 2, 4, and 5 of Table 10, respectively.

The correlation between *m* and $P_{critical}$ in those designs can be verified as shown in Figure 19.

It is worth mentioning the design presented in the second line of Table 10, whose mass is 1.707kg and $b_{I}>0$, confirms that the inverse problem is effectively solved in the discovery of feasible designs with reduced mass requirements. Designs with reduced mass values of 1.448kg and 1.690kg, and with positive post-buckling stiffness $b_{I}>0$, were also found in lines 12 and 13 of the table, respectively.

**Figure 22:**
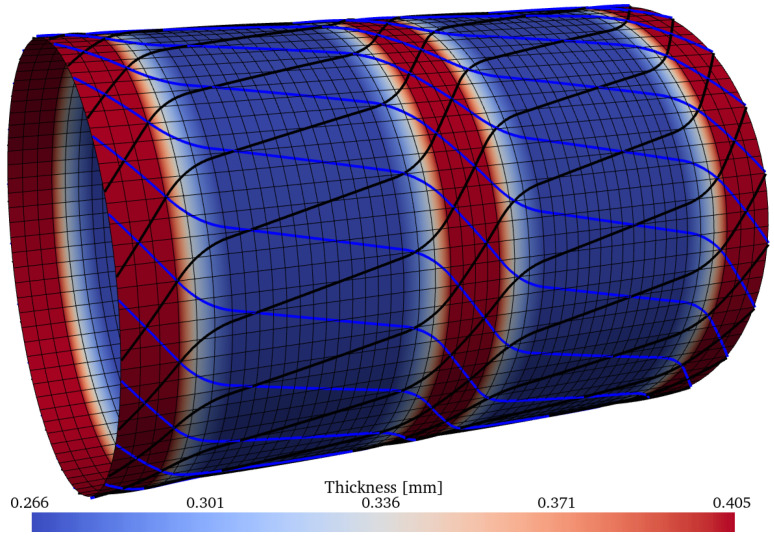
Optimum design ID = 12 from the Pareto front of Table 10, with m=1.448kg. The contour shows the thickness distribution. The black and blue lines represent, respectively, the CTS tow paths of each layer.

**Figure 23:**
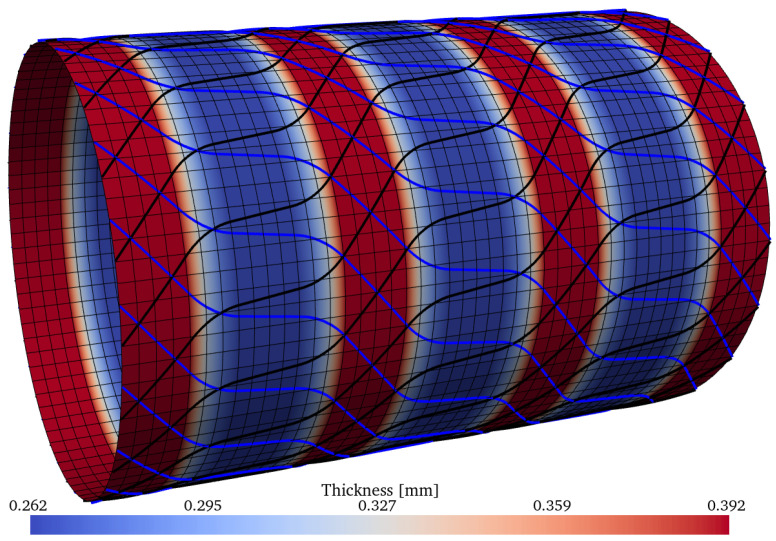
Optimum design ID = 1 from the Pareto front of Table 10, with m=1.510kg.

**Figure 24:**
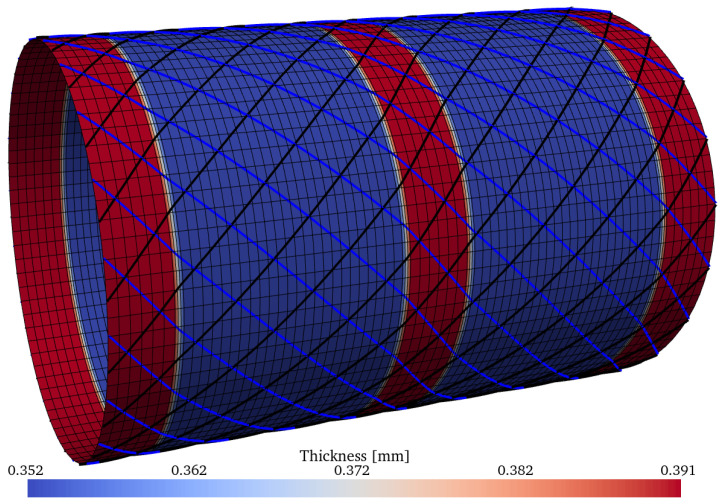
Optimum design ID = 13 from the Pareto front of Table 10, with m=1.690kg.

The three best optima of Table 10 have the following optimum mass values: m=1.448kg, m=1.510kg, and m=1.690kg; these are illsutrated, respectively, in Figure 22, Figure 23 and Figure 24. The thickness pattern of these optima have a close resemblance with the imperfection-insensitive hybrid shells proposed by Wagner et al. [77], where hoop-oriented belts were installed on top of a monolithic cylindrical shell to achieve the imperfection-insensitive behavior described by the authors. Furthermore, Lincoln et al. [78] also reported imperfection-insensitive designs with hoop-oriented thickness patterns created by CTS, and the encountered optima reported therein also resemble what the inverse ML-driven solver found in the present study. The nonlinear behavior of the optimum configuration with m=1.448kg was evaluated using the ABAQUS finite element solver using a mesh of 240 general-purpose elements S4R around the circumference, which have 4 nodes and reduced integration, while keeping the element aspect ratio close to 1. The results are compared against two constant-stiffness cases, where the cylinders were produced by setting $\theta_{1}=\theta_{2}$ and equal to $0^{\circ }$ and $45^{\circ }$, respectively. For the latter, the thickness of the plies is higher by a factor of $1/\cos45^{\circ }$ due to the nonlinear steering–thickness coupling produced by the CTS manufacturing process, as previously explained.

### Imperfection-Sensitivity Analysis

The imperfection sensitivity of the optimal design obtained after solving the aforementioned inverse problem is evaluated using single-perturbation load imperfections (SPLI) [79]. The performance is compared with two designs: one design named “Case $0^{\circ }$”, having two plies with all the fibers oriented parallel to the axial direction; and another named “Case $\pm 45^{\circ }$”, with two plies oriented at $\pm 45^{\circ }$. Note that, in the latter design, the thickness is increased by a factor of 1/cos45, assuming manufacturing by means of CTS. All analyses are performed using ABAQUS and 240 S4R elements around the circumference, as detailed in Castro et al. [80]. The name boundary conditions used for the optimization are herein realized by fixing the bottom- and top-edge nodes in the *y* and *z* directions, and by fixing the translation in the *x* direction for all nodes laying in the middle cross-section of the cylindrical shell. Note that this renders a load-controlled compression analysis, where the nodes at the edges are allowed to move separately from each other in the axial direction. The results of the imperfection sensitivity analysis are presented in Table 11, where the optimum cylinder herein obtained (design ID = 12 of Table 10) shows a reduced imperfection sensitivity when compared to Case $0^{\circ }$ and Case $\pm 45^{\circ }$.

In summary, a new methodology was proposed that encompasses modeling (Algorithm 1), training (Algorithm 2), and optimizing (Algorithm 3). Numerical computation confirmed the feasibility of the proposal, whose main advantages are:

- effective modeling strategy that explores the design space;
- uses popular machine learning methods without needing to adjust special parameters;
- optimization proposition that leads from the Design of Experiments (Figure 17) to optimal designs on the boundary of the compromise between mass and stiffness (Figure 19, Figure 20 and Figure 21).

Eighteen machine learning instances were evaluated on three datasets. No single strategy demonstrated superior performance in all cases (Figure 14, Figure 15 and Figure 16). On the other hand, sub-optimal machine learning strategies were also effective in supporting the optimization process. This confirms the need to select a reliable workflow (Figure 18) that will not degrade due to poor machine learning performance.

As a result, the authors believe that the present methodology constitutes a contribution to the design phase of a new project.

## 7. Conclusions

The present study proposed a design parameterization compatible with the continuous tow shearing (CTS) manufacturing process that is able to produce lightweight variable-stiffness cylindrical structures with tailored buckling load and reduced imperfection sensitivity.

The new methodology consists of contributions in the areas of problem modeling, the selection of machine learning strategies, and the proposition of an optimization formulation that results in optimal designs around the compromise frontier between mass and stiffness.

The parameterization developed herein took into account the nonlinear steering–thickness variation, which resulted in hoop-oriented regions of variable thickness for steering angles larger than zero. The maximum number of regions of larger thicknesses depends on the minimum fiber-steering radius, here assumed to be 50 mm, reportedly achievable by the CTS process.

An optimization goal to minimize the mass of cylindrical shells while keeping their post-buckling stiffness positive was proposed. The investigated problem consisted of a cylindrical shell under load-controlled axial compression, where both edges are simply supported and allowed to deform unevenly in the axial direction under the load application. The shells should be able to withstand a given design load, which is then compared with its linear buckling load. The post-buckling stiffness and linear buckling load are assessed by means of an enriched finite element model, using a formulation based on 10 degrees of freedom per node and a third-order interpolation of all the displacement field variables. For the post-buckling stiffness, a displacement-based formulation of Koiter’s method was adopted.

The machine learning models enabled a new design perspective based on the solution of a highly nonlinear inverse problem, and this proposition was effective in determining the input variables from a pre-determined design load. It is worth noting that all three of the methods performed well when using a suitable kernel choice, and the election of the best methodology would change if another criteria is considered, such as computational time or maximum error.

In addition to the quality of the designs provided by the methodology, as can be seen in Table 10, the formulation does not require additional parameter adjustments. Therefore, the investigation of the design space became straightforward.

Further studies will focus on applying Koiter’s method from a nonlinear pre-buckling state while looking for fully imperfection-insensitive shell designs that are capable of reaching post-buckled stiffness in load- and displacement-controlled applications. Furthermore, a detailed reliability-based evaluation of the obtained shells will be performed to map how robust the obtained positive post-buckling stiffness is in terms of angle and thickness imperfections, as well as variations in material properties. The evaluation of buckling behavior after an imperfection created post-impact, which was investigated, for instance, by Maziz et al. for filament-wound hybrid composite pipes [4], would be an interesting next step.

## Figures and Tables

**Table 1:** Error statistics of the $b_{I}$ meta-model using distinct SVM kernel options.

Id	Kernel	Min.	Median	Max.
1	Laplace	0.00000	0.00008	0.18026
2	RBF	0.00000	0.00012	0.18438
3	Poly	0.00000	0.00019	0.18768
4	Vanilla	0.00000	0.00019	0.18769
5	Tanh	0.00162	1.29576	6.39274
6	Bessel	0.00001	0.00341	0.18597
7	ANOVA	0.00506	0.61795	1.71422

**Table 2:** Error statistics of the mass estimate using distinct SVM kernel options.

Id	Kernel	Min.	Median	Max.
1	Laplace	0.00224	0.09454	14.09538
2	RBF	0.00020	0.12610	9.27851
3	Poly	0.00544	0.49763	529.40430
4	Vanilla	0.00628	0.49850	529.81813
5	Tanh	0.12038	41.58680	5921.04232
6	Bessel	0.00129	0.35197	339.96942
7	ANOVA	1.93005	28.31336	12,837.05073

**Table 3:** Error statistics of the $P_{critical}$ estimate using distinct SVM kernel options.

Id	Kernel	Min.	Median	Max.
1	Laplace	0.00020	0.11691	46.73723
2	RBF	0.00024	0.14848	35.81538
3	Poly	0.00210	0.62256	166.42386
4	Vanilla	0.00242	0.62194	166.51978
5	Tanh	0.04686	52.67757	16,211.19438
6	Bessel	0.00086	0.33308	87.76449
7	ANOVA	0.17326	19.83168	5415.23160

**Table 4:** Error statistics of the $b_{I}$ meta-model using a distinct Kriging covariance kernel structure.

Id	Kernel	Min.	Median	Max.
1	Gauss	0.00000	0.00005	0.18798
2	Matern $5_{2}$	0.00000	0.00010	0.18809
3	Matern $3_{2}$	0.00000	0.00015	0.17966
4	Exp	0.00000	0.00033	0.18004
5	Powexp	0.00000	0.00080	0.18080

**Table 5:** Error statistics of the mass estimate using a distinct Kriging covariance kernel structure.

Id	Kernel	Min.	Median	Max.
1	Gauss	0.00004	0.01341	13.12694
2	Matern $5_{2}$	0.00001	0.02327	3.54187
3	Matern $3_{2}$	0.00010	0.02933	6.50619
4	Exp	0.00036	0.05714	20.58523
5	Powexp	0.00003	0.01734	22.41985

**Table 6:** Error statistics of the $P_{critical}$ estimate using a distinct Kriging covariance kernel structure.

Id	Kernel	Min.	Median	Max.
1	Gauss	0.00008	0.06522	43.00029
2	Matern $5_{2}$	0.00016	0.05478	39.23206
3	Matern $3_{2}$	0.00007	0.05416	36.73150
4	Exp	0.00011	0.05613	22.85848
5	Powexp	0.00042	0.05325	20.13985

**Table 7:** Error statistics of the $b_{I}$ meta-model using a Random Forest with distinct number of trees and splits.

Id	Tree/Split	Min.	Median	Max.
1	50/3	0.00000	0.00012	0.18004
2	100/3	0.00000	0.00013	0.18000
3	200/3	0.00000	0.00015	0.22901
4	50/5	0.00000	0.00005	0.31076
5	100/5	0.00000	0.00005	0.30622
6	200/5	0.00000	0.00007	0.27966

**Table 8:** Error statistics of the mass estimate using a Random Forest with distinct number of trees and splits.

Id	Tree/Split	Min.	Median	Max.
1	50/3	0.00001	0.09256	93.12868
2	100/3	0.00041	0.08894	81.82169
3	200/3	0.00026	0.08498	107.73466
4	50/5	0.00034	0.08707	77.93849
5	100/5	0.00003	0.08891	72.21142
6	200/5	0.00012	0.08465	94.27213

**Table 9:** Error statistics of the $P_{critical}$ estimate using a Random Forest with a distinct number of trees and splits.

Id	Tree/Split	Min.	Median	Max.
1	50/3	0.00018	0.07656	31.20040
2	100/3	0.00024	0.08051	16.20204
3	200/3	0.00004	0.07359	24.21144
4	50/5	0.00002	0.08778	23.46504
5	100/5	0.00007	0.08512	31.84951
6	200/5	0.00016	0.08478	20.88944

**Table 10:** Design values obtained from the analysis of the inverse problem.

Id	$v_{1}$	$v_{2}$	$v_{3}$	$v_{4}$	$v_{5}$	*m*	$P_{\mathrm{critical}}$	$b_{I}$
1	0.1199	3	0.4123	48.41	7.60	1.510	4885	−0.004
2	0.1487	4	0.4377	47.44	41.43	1.707	8024	0.038
3	0.0953	5	0.5615	49.68	51.61	1.910	11,344	−0.003
4	0.1545	5	0.6250	54.75	56.16	2.139	14,253	−0.002
5	0.1500	5	0.5299	59.09	57.71	2.303	17,020	−0.006
6	0.1524	4	0.5205	60.55	62.40	2.534	20,467	−0.002
7	0.1520	4	0.4729	65.19	62.33	2.745	23,818	0.031
8	0.1520	4	0.5200	67.06	63.55	2.896	25,779	0.035
9	0.0885	8	0.4050	67.56	67.00	3.133	31,053	0.342
10	0.1582	4	0.5127	66.79	70.41	3.338	34,298	0.095
11	0.1217	4	0.4316	68.98	70.37	3.465	37,080	0.309
12	0.1405	2	0.6810	50.10	12.63	1.448	4758	0.095
13	0.1450	2	0.7022	48.38	42.34	1.690	8151	0.033
14	0.1575	4	0.5750	49.95	51.61	1.915	11,375	−0.003
15	0.1520	5	0.5999	54.75	57.01	2.166	14,739	−0.002
16	0.1520	5	0.6650	59.09	57.76	2.293	16,918	−0.007
17	0.1525	6	0.6654	63.68	57.98	2.435	18,751	−0.005
18	0.1805	4	0.4348	66.48	61.05	2.788	23,240	−0.017
19	0.1617	4	0.4436	64.22	68.53	3.009	27,982	0.029
20	0.1043	6	0.4595	65.79	70.51	3.254	31,232	0.005
21	0.1632	4	0.4677	69.07	68.50	3.340	34,555	0.350
22	0.1624	4	0.4705	70.86	68.51	3.500	36,835	0.317

**Table 11:** Imperfection sensitivity of Case $0^{\circ }$, Case $\pm 45^{\circ }$, and the design optimum ID = 12 from Table 10.

	Pristine	BL (N)	BL (N)	KDF	KDF
	Buckling	with a	with a	with a	with a
	Load (N)	SPLI of 0.1N	SPLI of 1.0N	SPLI of 0.1N	SPLI of 1.0N
Case $0^{\circ }$	3017	1747	225	57.9%	7.4%
Case $\pm 45^{\circ }$	4261	3402	2040	79.8%	47.9%
Optimum Id = 12	3017	2511	1831	83.2%	60.7%

## Data Availability

The DOE data supporting the present study is available in a public dataset [61].
